# Cavemen Were Better at Depicting Quadruped Walking than Modern Artists: Erroneous Walking Illustrations in the Fine Arts from Prehistory to Today

**DOI:** 10.1371/journal.pone.0049786

**Published:** 2012-12-05

**Authors:** Gabor Horvath, Etelka Farkas, Ildiko Boncz, Miklos Blaho, Gyorgy Kriska

**Affiliations:** 1 Department of Biological Physics, Physical Institute, Eotvos University, Budapest, Hungary; 2 Department of Physics, Institute of Mathematics and Physics, Savaria Campus University of West Hungary, Szombathely, Hungary; 3 Group for Methodology in Biology Teaching, Biological Institute, Eotvos University Budapest, Hungary; University of Western Ontario, Canada

## Abstract

The experts of animal locomotion well know the characteristics of quadruped walking since the pioneering work of Eadweard Muybridge in the 1880s. Most of the quadrupeds advance their legs in the same lateral sequence when walking, and only the timing of their supporting feet differ more or less. How did this scientific knowledge influence the correctness of quadruped walking depictions in the fine arts? Did the proportion of erroneous quadruped walking illustrations relative to their total number (i.e. error rate) decrease after Muybridge? How correctly have cavemen (upper palaeolithic *Homo sapiens*) illustrated the walking of their quadruped prey in prehistoric times? The aim of this work is to answer these questions. We have analyzed 1000 prehistoric and modern artistic quadruped walking depictions and determined whether they are correct or not in respect of the limb attitudes presented, assuming that the other aspects of depictions used to determine the animals gait are illustrated correctly. The error rate of modern pre-Muybridgean quadruped walking illustrations was 83.5%, much more than the error rate of 73.3% of mere chance. It decreased to 57.9% after 1887, that is in the post-Muybridgean period. Most surprisingly, the prehistoric quadruped walking depictions had the lowest error rate of 46.2%. All these differences were statistically significant. Thus, cavemen were more keenly aware of the slower motion of their prey animals and illustrated quadruped walking more precisely than later artists.

## Introduction

Some important details of quadruped walking are known since the pioneering work of Eadweard Muybridge [1], [2], [3]. Thus since the 1880s scientists know how horses and other tetrapods walk. Due to the comprehensive studies of different gaits of numerous quadruped species the knowledge on quadrupeds’ locomotion became enormous [4], [5], [6], [7], [8], [9], [10], [11], [12], [13], [14], [15], [16], [17]. The experts of animal locomotion are well aware of the fact, for example, that the majority of quadrupeds advance their legs in the same sequence when walking, and only the timing of the various combinations of supporting feet differ more or less. The usual succession of the ground-contacting feet of walking quadrupeds, termed as foot-fall formula, is -LH-LF-RH-RF-, where L/R and H/F mean left/right and hind/fore, respectively. This is known as the lateral sequence walk. The reason for this uniformity of walking is that the body of quadrupeds has maximal stability when using this gait, therefore it is preferred by many animals. Some quadrupeds (mainly primates) use a diagonal sequence walk, but the lateral sequence walk is much more common.

One could assume that the time period of more than 120 years ellapsed since the publications of Muybridge [1], [2], [3] might have been long enough for taxidermists of natural history museums, animal anatomists and designers of animal toy models to learn how quadrupeds walk. It was, however, shown that this is not the case [18], [19]. If the number of correct and incorrect quadruped walking illustrations is *N*_correct_ and *N*_incorrect_, respectively, their error rate is defined as *r* = *N*_incorrect_/(*N*_correct_+*N*_incorrect_). The error rates of the quadruped walking depictions investigated by Horvath and co-workers [18], [19] were the following: (i) *r*_museum_ = 41.1% for natural history museums. (ii) *r*_taxidermy_ = 43.1% for taxidermy catalogues. (iii) *r*_book_ = 63.6% for animal anatomy text-books. (iv) *r*_toy_ = 50% for quadruped toy models. (v) *r*_total_ = 46.6% for the total 307 studied quadruped walking depictions.

The leg attitudes of walking quadrupeds, especially horses, are also frequently erroneously illustrated in the works of fine arts [20], [21]. These artistic representations of walking quadrupeds have not been systematically studied from a biomechanical point of view. To fill this gap, we have collected 1000 different fine art quadruped walking illustrations from the Internet and other sources. We analysed them to decide whether they are correct or not in respect to the relative limb positions with the assumption that the other aspects of statues, paintings, drawings and reliefs used to determine the animals gait are depicted correctly. As a result we have determined the rate *r* of erroneous artistic quadruped walking depictions. We obtained the error rates of artistic quadruped walking illustrations for the prehistoric period, for the pre-Muybridge time (after prehistory but prior to 1887) and for the post-Muybridge period (after 1887). We have also calculated the error rate for three-dimensional (cavalry statues) and two-dimensional (paintings, graphic art, reliefs) artistic quadruped walking depictions. Some preliminary results have been published in a Hungarian popular journal [22].

## Results

**Figure 1:**
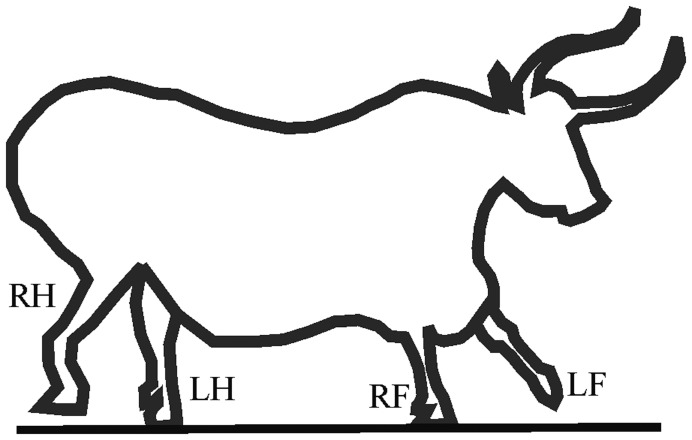
Contour of a bull copied from a picture of a prehistoric painting in the French cave Lascaux. (The original colour picture can be found in the following website: http://pittkyle123.wordpress.com/2011/02/15/cave-paintings-30000-years-ago). The straight line represents the assumed ground line. LH: left hind leg, LF: left fore leg, RH: right hind leg, RF: right fore leg. This correct quadruped walking illustration falls in the cell *Bb* of the walking matrix.

Figure 1 shows a prehistoric picture of a bull from the French cave Lascaux, where the straight line represents the assumed ground line. The LH and RH legs of this bull are on the ground, the RH leg is lifted, and the LF leg has just been placed on the ground. This correct quadruped walking illustration falls in the cell *Bb* of the walking matrix.

**Figure 2:**
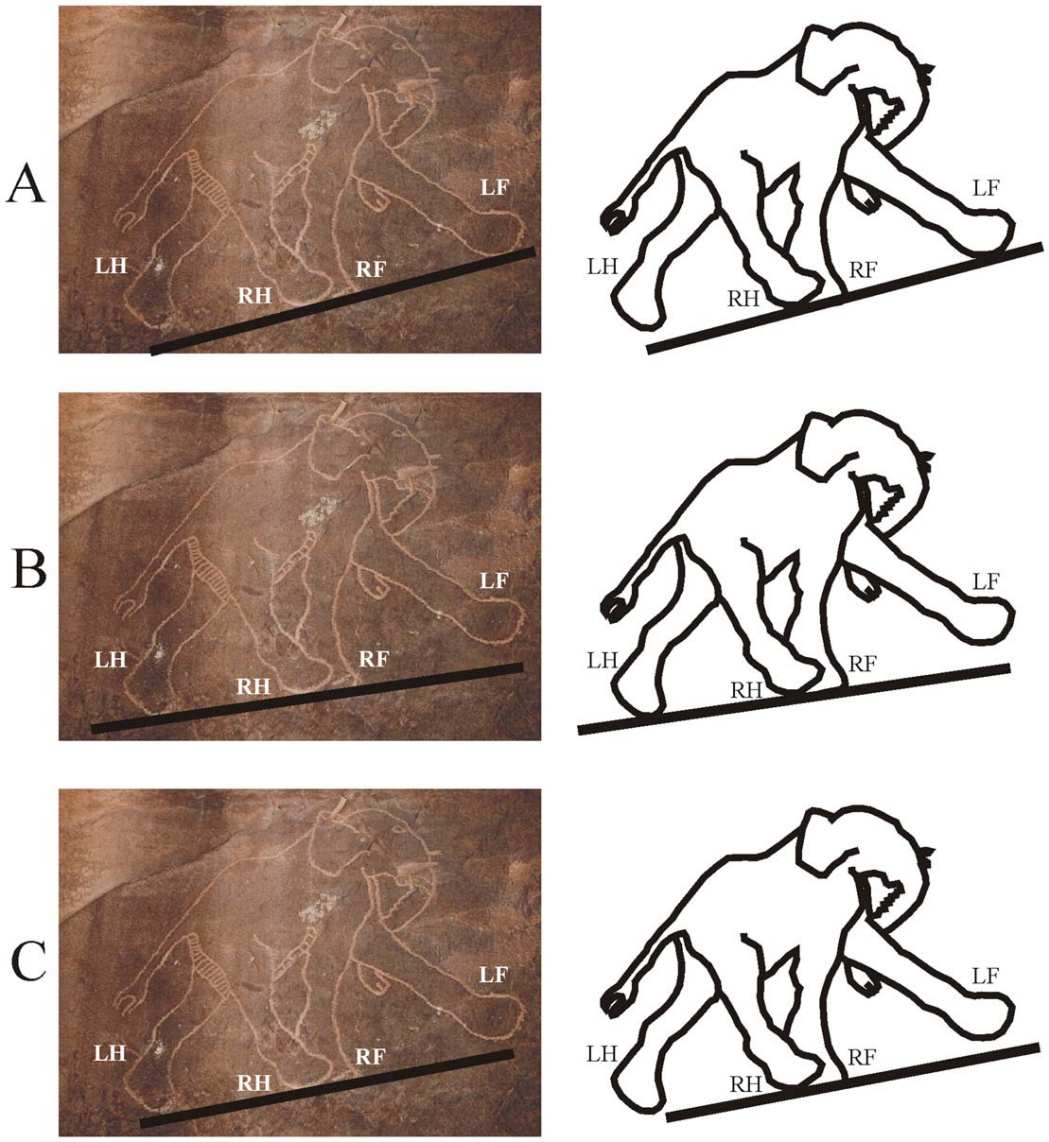
A prehistoric elephant depiction. *Left*: Prehistoric illustration of an elephant from the Libian Tadrart Acacus (http://www.galuzzi.it, the permission from the photographer, Luca Galuzzi is found in the Supporting Online Material). *Right*: Contour of the elephant. Here there are three possibilities (A, B, C) for the alignment of the assumed ground line: In cases (A), (B) and (C) the walking depiction falls in cells *Be*, *Cf* and *Bf* of the walking matrix, respectively, all being incorrect.

In Fig. 2 a prehistoric picture of an elephant from the Libian Tadrart Acacus can be seen. In this case there are three possibilities, depending on the alignment of the assumed ground line: (i) In Fig. 2A the LH, RH and RF legs are on the ground, and the LF leg is in its falling phase. (ii) In Fig. 2B the LF leg has just been lifted, while the RH, RF and LF legs are on the ground. (iii) In Fig. 2C the RH and RF legs are in contact with the ground, the LH leg is lifted, and the LF leg has just been placed onto the ground. In cases (i), (ii) and (iii) the walking depictions fall in cells *Be*, *Cf* and *Bf* of the walking matrix, respectively, all being incorrect.

**Figure 3:**
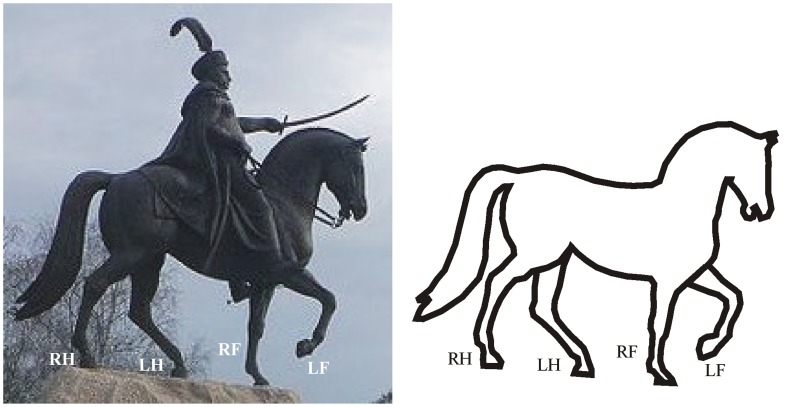
A correct modern, post-Muybridgean cavalry statue (Lajos Győrfi: cavalry sculpture of the Polish king Jan Sobieski III., Tatabanya, Hungary), the walking depiction of which fits into the cell *Ba* of the walking matrix. *Left*: Picture of the statue (photo taken by Gabor Horvath). *Right*: Schematic drawing of the horse.

**Figure 4:**
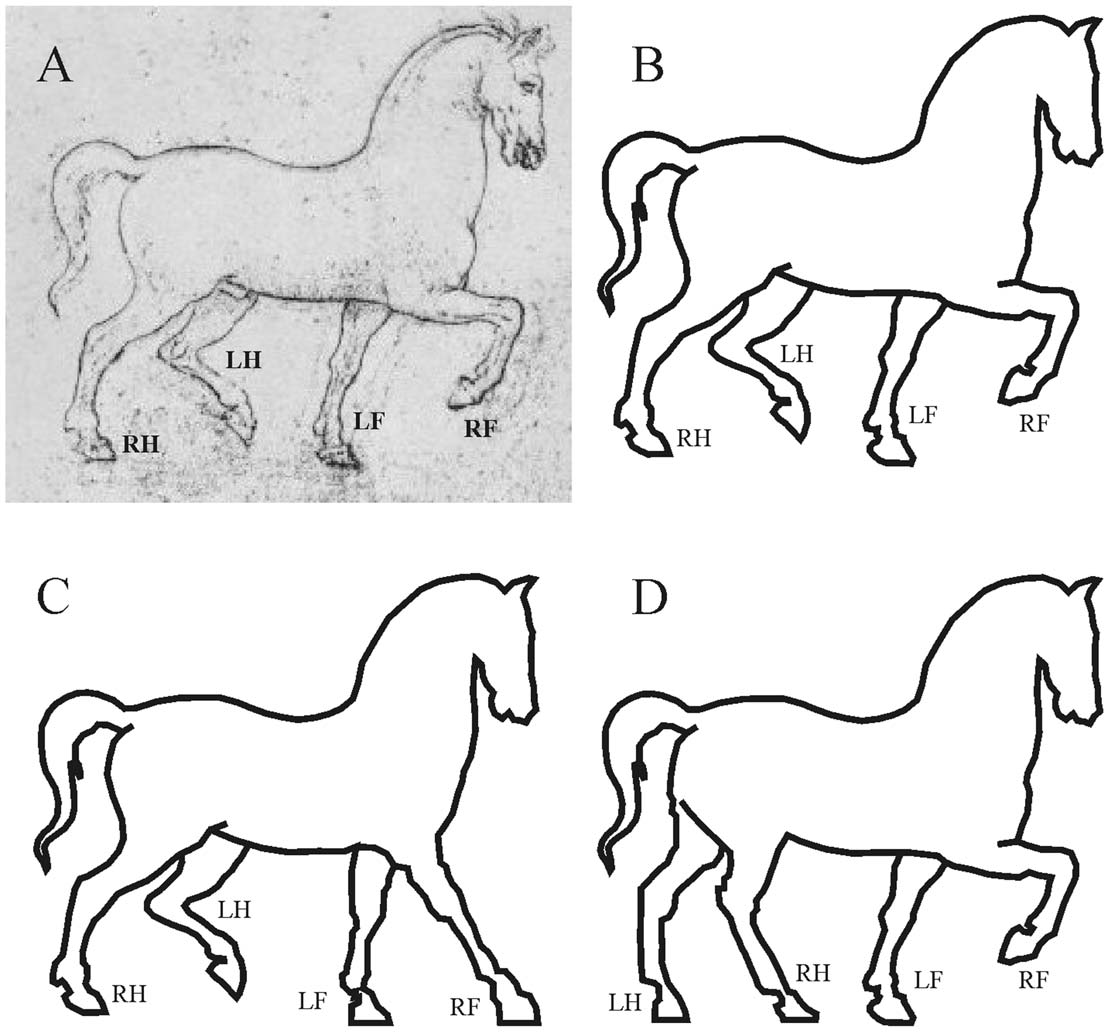
An erroneous modern, pre-Muybridgean horse drawing of Leonardo da Vinci (http://www.davincisketches.com). (A, B) The erroneous horse drawing fits into the cell *Eh* of the walking matrix. (A) Picture of the graphic art. (B) Schematic drawing of the horse. (C, D) Two possible corrections of the horse: C keeps the postures of the hind legs and corrects the attitudes of the fore legs, thus falls into the cell *Gh* of the walking matrix. D, keeping the postures of the fore legs and correcting the attitudes of the hind legs, belongs to the cell *Ee* of the walking matrix.

In Fig. 3 the cavalry statue is post-Muybridgean and its correct walking depiction fits into the cell *Ba* of the walking matrix. In Figs. 4A and 4B an incorrect pre-Muybridgean horse drawing by Leonardo da Vinci can be seen fitting into cell *Eh*. Figures 4C and 4D demonstrate how the error of horse leg postures could be corrected, if the hind leg attitudes are kept and the fore leg postures are corrected (Fig. 4C, falling into the cell *Gh* of the walking matrix), or the fore leg postures are kept and the hind leg attitudes are corrected (Fig. 4D, fitting into cell *Ee* of the walking matrix). Of course, there are many other correction possibilities. We have presented here those two corrections that are closest to the original quadruped walking illustrations.

Table 1 shows the number (*N*) of the different quadruped walking depictions analyzed, the numbers of correct (*N*_correct_) and incorrect (*N*_incorrect_) depictions, their error rates *r* = *N*_incorrect_/(*N*_correct_+*N*_incorrect_), furthermore the tables containing the numbers of the analyzed illustrations in the walking matrix. The distribution of the modern and prehistoric quadruped walking depictions in the walking matrix is seen in Tables S1 and S2. Tables S3, S4, S5, S6, S7 and S8 show the numbers of correct and incorrect prehistoric plus modern, pre-Muybridgean, post-Muybridgean, 2-dimensional (cavalry statues), 3-dimensional (paintings, graphic art, reliefs) and horse walking illustrations in the walking matrix. Table S9 is the unity walking matrix with number 1 in its every cell. According to Table 2, all differences between the various quadruped walking depictions are statistically significant. There was, however, no significant difference between the error rates of 3-dimensional (cavalry statues: 65.5%) and 2-dimensional (paintings, graphic art, reliefs: 65.1%) depictions.

**Table 1:** Number (*N*) of the different quadruped walking depictions analyzed, numbers of correct (*N*_correct_) and incorrect (*N*_incorrect_) depictions, their error rates (*r*) and tables of the walking matrices.

quadruped walking depiction type	number (*N*)	correct (*N*_correct_)	incorrect (*N*_incorrect_)	error rate (*r*)	walking matrix
modern	961	334	627	65.2%	Table S1
prehistoric	39	21	18	46.2%	Table S2
total (prehistoric+modern)	1000	355	645	64.5%	Table S3
modern, pre-Muybridgean	272	45	227	83.5%	Table S4
post-Muybridgean	686	289	397	57.9%	Table S5
cavalry statues (3D)	359	124	235	65.5%	Table S6
paintings, graphic art, reliefs (2D)	602	210	392	65.1%	Table S7
horse walking (prehistoric and modern)	829	244	585	70.6%	Table S8

**Table 2:** Statistical comparisons (binomial χ^2^ test) between the numbers (*N*_incorrect_) of incorrect quadruped walking illustrations (Table 1) to test differences between various ages (modern, post-Muybridgean, pre-Muybridgean, prehistoric), 2- and 3-dimensional depictions, and the random choice.

comparison of two depiction groups	?^2^	d*f*	*p*	significance
**various ages compared with the random case**				
prehistoric *versus* random	14.70	1	0.0001	significant
pre-Muybridgean *vs*. random	14.33	1	0.0002	significant
post-Muybridgean *vs*. random	83.441	1	<0.0001	significant
modern *vs*. random	31.86	1	<0.0001	significant
**various ages compared with each other**				
pre-Muybridgean *vs*. prehistoric	151.9	1	<0.0001	significant
post-Muybridgean *vs*. pre-Muybridgean	327.0	1	<0.0001	significant
post-Muybridgean *vs*. prehistoric	37.6	1	<0.0001	significant
modern *vs*. prehistoric	140.2	1	<0.0001	significant
**3-dimensional compared with 2-dimensional**				
3D (cavalry statues) *vs*. 2D (paintings, graphic art, reliefs)	<0.01	1	0.987	not significant

For the random case the unity walking matrix has number 1 in its every cell (Table S9). Then the numbers of correct and incorrect quadruped walking illustrations are: *N*_correct_ = 16, *N*_incorrect_ = 44, total *N* = *N*_correct_+*N*_incorrect_ = 60, resulting in an error rate of *N*_incorrect_/*N* = 73.3% corresponding with the pure accident.

## Discussion

On the basis of the relative leg positions one could assume that the elephant in Fig. 2 is trotting. However, trot is a running sequence, and elephants generally do not run. Although Asian elephants (*Elephas maximus* L.) can move at speeds up to 25 km/h and at this maximal speed some features of their locomotion conform to certain definitions of running [23], their usual motion is walking. Although the fastest gait used by elephants has been variously described as a walk, amble, trot, pace, rack or a running walk, even fast moving elephants maintain the same walking foot-fall pattern (lateral sequence: -LH-LF-RH-RF-) and always keep at least one foot in contact with the ground [23]. Consequently, elephants do not trot. This is the reason why we considered the picture in Fig. 2 as a walking depiction, rather than a trotting sequence. On the other hand, Fig. 2 would be a correct motion depiction, if it represented a trotting elephant. In this case the error rate of prehistoric walking depictions would decrease, which could strengthen our main conclusion, that prehistoric walking depictions are more accurate than the modern ones.

On the basis of the leg attitudes Fig. 4A could, in principle, depict a trotting horse. However, because the fore legs of trotting horses are never lifted so high, and the angle between the *femur* and *tarsus* cannot be nearly 90° this should be a walking horse. This is evident from the series of pictures taken of trotting horses by Muybridge (1887), for example.

The horse leg poses of cavalry statues are often symbolic: an elevated right forefoot, for instance, might indicate that the rider (e.g. a general) died in combat [21]. Such symbolic depictions can result in erroneous walking illustrations. We admit that, of course, in our present work there is some speculation, because we could not ask the prehistoric or modern artists why they have composed certain drawings and depicted quadrupeds in a particular way. We presented here an optimal and simple way to compare one aspect, namely the accuracy of quadrupeds in a walking mode of locomotion. In this respect, it was irrelevant whether the artists’ intention was to show an animal in a natural or unnatural pose.

The depiction of animals dates back to the prehistoric era, when people used cavepaintings and carvings to illustrate the animals they hunted. Since the observation of animals was not merely a pastime, but a matter of survival, we can suppose that compared to artists of latter eras, when people were not as directly connected to nature, the creators of such cavepaintings and carvings observed their subjects better and thus they depicted the walk of the animals in a more life-like manner. This is, in fact the conclusion of our examinations.

**Figure 5:**
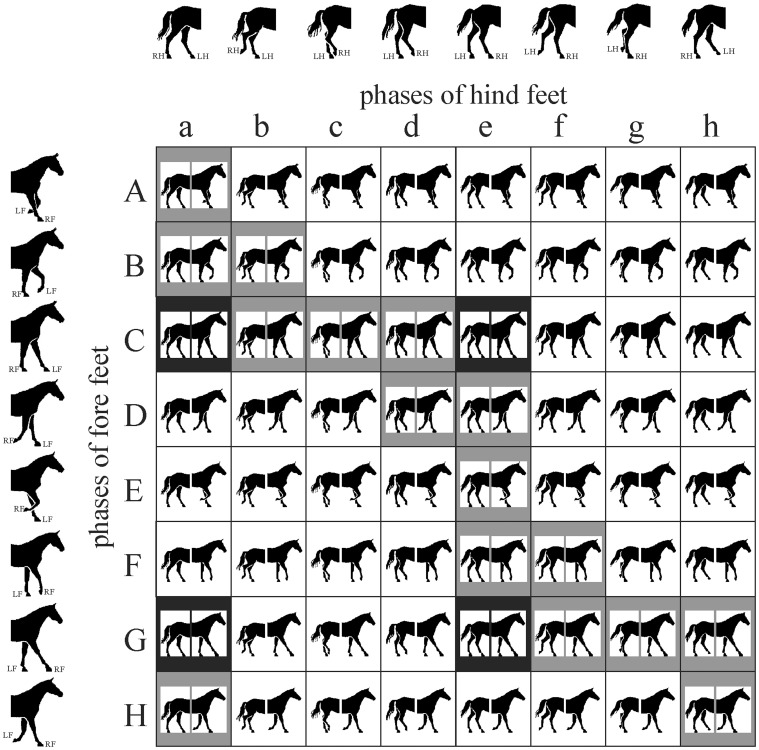
The 8×8 walking matrix of the stride of the horse walking from left to right. To each of the 8 columns and 8 rows belong given postures of the hind and fore feet pair, respectively, as shown by the black half horse contours on the top and left border. These horse contours coincide with the scientific drawings of the eight phases of the stride of walking horses published by Gambaryan [11], [12]. In a given cell of the matrix the fore feet attitudes belonging to the cell’s row are paired with the hind feet postures belonging to the cell’s column.

The likelihood of pure chance can be calculated from the unity walking matrix with a single case in its every cell: In this case the numbers of correct (grey cells) and incorrect (white cells) quadruped walking illustrations are *N*_correct_ = 16 and *N*_incorrect_ = 44, resulting in an error rate of *r* = *N*_incorrect_/(*N*_correct_+*N*_incorrect_) = 73.3% corresponding with mere chance. Hence if an artist chose purely randomly the leg postures in a quadruped walking illustration, that is selected randomly from the walking matrix (Fig. 5), then the leg attitudes would be erroneous with a chance of 73.3%.

The lowest rate of error in quadruped walking illustrations analized by us, was found in cave art 46.2%, which is close to the value found by Horvath *et al*. [19] having examined the walking illustrations in natural history museums, anatomy text-books and toy figurines (46.6%). This near 50% value does not mean that prehistoric men illustrated quadruped motion by chance as we have just stated. Compared to the 73.3% value the 46.2% rate of error is fairly low, which shows that 53.8% of the prehistoric illustrations are correct and that prehistoric man was an apt observer. This is understandable since their hunting lifestyles were strongly dependent on the quadruped animals they hunted, and prehistoric artists might merely depicted the animals as they observed them during hunting.

The 46.2% error rate of the prehistoric quadruped walking illustrations is nearly half of the 83.5% error rate of the pre-Muybridgean illustrations. This is surprising, since it could be justly expected that the prehistoric man, with a primitive culture and artistic techniques, would work with a much greater rate of error than his later counterparts. Prehistoric men illustrated the walking of quadrupeds with almost the same error rate (46.2%) as the taxidermists of natural history museums (41.1−43.1%) [19].

The 65.2% error rate of the walking quadruped illustrations dating from after prehistoric times is only 8.1% less then the 73.3% of illustrating randomly. Therefore we can say that the historic artists in effect, merely illustrated the walking of quadrupeds by chance. Prior to the works of Muybridge but during the historic era the error rate in depicting quadruped motion was 83.5%, whereas after Muybridge this value decreased to 57.9%. This 25.6% decrease is most logically attributed to the positive effect of Muybridge’s work and the spread of photography on the artistic community. The post-Muybridgean 57.9% error rate is approximately that of the 63.6% error rate of the animal anatomy text-books [19], probably because these text-books also date from after Muybridge.

Interestingly, before Muybridge, the 83.5% error rate is greater than the accidental 73.3%. Hence, the artists predating Muybridge did not illustrate the walking of quadrupeds by chance, instead they might depicted quadrupeds possibly by mimicking earlier, erroneous works.

The rate of error in the depiction of walking in horse-statues is 65.5%, which is practically tha same as the error rate of 65.1% in paintings, drawings and reliefs. However, the most commonly occuring error in horse-statues is the cell *Bd* cell in the walking-matrix, whilst in paintings, drawings and reliefs the most common error falls in the cell *Eh*. The reason for this may be that quadrupeds depicted with erroneous walking in paintings, drawings and reliefs cannot fall over. On the other hand, an error in the leg positioning can substantially reduce the static stability of an entire horse-statue. In this way, certain errors cannot occur in the sculpting of horse-statues.

We found that modern artists err considerably more often (65.2%) in horse-walk depictions than do taxidermists, anatomy text-book writers and toy figurine designers (50.4%) [19].

In this work we showed that prehistoric men (upper palaeolithic *Homo sapiens* called simply as “caveman” in this work) depicted quadruped walking more correctly than modern artists. We admit that it is difficult to scientifically assess this surprising fact. It would be difficult to perform a really fair comparison between (prehistoric and modern) artistic quadruped walking illustrations and the real walk of living quadrupeds, because there is no proof that the investigated examples of modern art intended to represent walking in a standard way. Being paintings or sculptures, for instance, these are static poses of whatever motion the artists wanted to express, not necessarily a standard walk. Here we tried to study this problem as correctly as possible: We disregarded any hypothetical or speculative artistic aim, and compared the leg attitudes of quaruped walking depictions in the fine arts with those of the real walking gaits of horses. As results, we obtained raw numbers of the incorrect and correct artistic walking illustrations, from which our final message, the error rates were derived for different (prehistoric, pre- and post-Muybridgean) epochs. This is the maximum of what can scientifically be done in this topic.

## Materials and Methods

### Ethics Statement

No specific permits were required for the studies described here. No specific permissions were required for these locations and activities. The studies did not involve endangered or protected species.

### Collecting Quadruped Walking Illustrations

The majority of the analyzed quadruped walking illustrations were collected from the Internet (some most important websites were: www.bradshawfoundation.com, en.wikipedia.org, www.equineartists.co.uk, www.szoborlap.hu, www.lascaux.culture.fr, www.hermitagemuseum.org, www.fitzmuseum.cam.ac.uk). Additionally, we found several such depictions in books of fine art, furthermore we photographed numerous cavalry sculptures paintings, graphic arts and reliefs in different Hungarian cities and museums of fine art. We had also found numerous walking horse illustrations on stamps and coins. Only those quadruped walking illustrations were analysed, in which the animals lifted one or two legs, so that it had 2 or 3 supporting feet. Depictions with 4 supporting feet were not considered. Finally, in total we have analysed *N* = 1000 two- and three-foot-supported quaruped walking depictions. For the sake of an easier analysis, we aligned (mirrored if necessary) all collected pictures in order to make the animal walk from left to right. Since the quadruped walking illustrations were gathered randomly and independently from each other, the conclusions drawn from them can be considered statistially correct. Thus a further increase of *N* would not result in considerably different results and conclusions.

During the selection of these quadruped motion depictions we excluded images representing non-walking gaits. Since there are no information about the original intention and artistic license of artists, it was impossible to decide whether artists depicted correctly or erroneously the angle of tail, positions of the mane, neck and head, and other aspects on the illustrations. Thus, we concentrated on the biomechanically primary variable, the foot-fall pattern, and assumed that the other, biomechanically secondary aspects of statues, paintings, drawings and reliefs used to determine the animals gait are depicted correctly. The logical reason for this assumptions is the following: It is difficult, if not impossible, to observe with the naked eye the exact foot-fall pattern of quadrupeds, especially at quicker motions, such as trotting and running. Much easier is to observe the angle of tail and the positions of the mane, neck and head, furthermore the details of the rider. Consequently, it is more probable that an artist depicted the leg attitudes of an animal erroneously and correctly illustrated the other aspects. If a rider or other human artifact was not present in an illustration, there remained still enough descriptors (angle of tail, mane position, neck direction, head holding) to discern the most probable gait of the animal.

We could distinguish unambiguously between walking and non-walking (running) representations on the basis of the following typical features: (i) ***Attitude of the tail and mane of the quadruped***: If the tail and/or the mane of a quadruped hung nearly vertically (maximum ±15° from the vertical) due to gravitation, the animal was considered walking, otherwise (for oblique tail/mane direction due to the drag) running was assumed. (ii) ***Attitude of the neck and head of the quadruped***: Steep angles (greater than 45°) of the neck and head of the quadruped relative to the horizontal indicated walking, while flat angles (smaller than 45°) indicated running. (iii) ***Folds of the rider’s mantle/coat and direction of the strap of the rider’s bag/case***: Nearly vertical (maximum ±15° from the vertical) folds of the rider’s mantle/coat and the strap of bag/case demonstrated the lack of drag, indicating a walking motion of the horse. In the case of running horses the mantle/coat fluttered in the wind, and the bag/case strap was oblique (>±15° from the vertical). (iv) ***Attitude of the rider’s body***: Straight and nearly vertical (maximum ±25° from the vertical) body attitude of the rider indicated a walking horse, since a rider could not sit straight and almost vertically on a running horse. (v) ***Additional information from the situation and surrounding***: Frequently, the depicted situation and surrounding referred to a slow motion (walking) of the quadruped. For, example, if a quadruped pulled a heavy wagon, or a rider proceeded majestically through a crowd of standing or walking people, the walking of the animal was logically assumed. The mentioned angles (±15°, ±25° and 45° from the vertical) originated from our survey: We collected 50 and 50 different photographs taken about walking and running horses with and without riders. In these photos the mentioned angles were measured, and their extrema were used.

In principle, it is impossible to determine whether the foot-fall pattern or the above-mentioned other aspects (position and direction of the tail, neck, head, mane, posture of the rider, etc.) on a given quadruped walking depiction are incorrect. This, however, is not a serious problem, because the depiction is wrong in both cases. Consequently, the error rate does not change. We assumed that the other aspects are correct and determined the accuracy (correct or incorrect) of the foot-fall pattern. If we changed our method, considering the foot-fall pattern always correct and studying the accuracy of the other aspects, the result would be the same: If the other aspects are inconsistent with the foot-fall pattern, the depiction is erroneous, otherwise it is correct.

### Analysis of Modern Quadruped Walking Illustrations

To analyse walking illustrations of all possible quadruped species we used the easy and reliable method developed by Horvath and co-workers [18], [19]. Since this method has been published only in a Hungarian text-book [18] and as an electronic supplementary material [19], below we describe it in detail.

There are maximum 8 phases (or combinations of support) in each locomotor cycle (stride) of quadrupedal walking [10]. We took the scientific drawings of these eight phases of the stride of walking horses (originating from the monographs of Gambaryan [11], [12]) and split them along a vertical axis into a fore and a hind half. Thus we obtained eight fore halves designated *A*, *B*, *C*, *D*, *E*, *F*, *G*, *H*, and eight hind halves designated *a*, *b*, *c*, *d*, *e*, *f*, *g*, *h* as seen in Fig. 5. All fore halves were paired with all hind halves in all possible ways, which resulted in the so-called “walking matrix” possessing 8 rows and 8 columns. Each cell of this matrix represents a theoretically possible quadruped walking illustration, in which the positions of the fore and hind feet are the same as those of the corresponding real phases of the stride of the walking horse (Fig. 5) belonging to the cell’s row and column, respectively. A cell of this matrix is designated by the capital letter of its row and the small letter of its column: cell *Bb*, for example, represents a correct quadruped walking depiction, because it corresponds to phase *B* of walking (Fig. 5), while cell *Db* is an imaginable but incorrect quadruped walking illustration, because it never occurs during real quadruped walking (i.e., the fore feet attitudes of phase *D* (Fig. 5) are never combined with the hind feet postures of phase *B* (Fig. 5).

The cells of the walking matrix (Fig. 5) with black and grey background represent those phases of the stride of (very slow, slow, or rapid) walkings, which are characteristic to all quadrupeds investigated until now [1], [2], [3], [10], [11], [12], when the foot-fall formula is -LH-LF-RH-RF-. We have considered a given two- and three-foot-supported quadruped walking depiction as correct only, if it correlates to the grey cells of the walking matrix (Fig. 5). Quadruped walking illustrations in the cells of the walking matrix with a white background were considered as incorrect, because they do not correspond to the foot-fall formula -LH-LF-RH-RF- of walking, that is they do not occur in the stride during walking.

In order to analyse correctly the postures of the left and right legs of both the fore and hind leg pairs relative to each other, during the analysis of quadruped walking depictions we referred to three main phases of the stride: lifting, swinging and falling. On the basis of these three leg phases, we sought the cell of the walking matrix, which best approximates the leg attitudes of a given quadruped walking illustration. Thus, the position (row, column) of every quadruped walking depiction was determined in the walking matrix.

### Analysis of Prehistoric Quadruped Walking Illustrations

During the detailed analysis of prehistoric quadruped walking depictions (see Supporting Informations S1 and S2, furthermore Figures S1, S2, S3, S4, S5, S6, S7, S8, S9, S10, S11, S12, S13, S14, S15, S16, S17, S18, S19, S20, S21, S22, S23, S24, S25, S26, S27, S28, S29, S30, S31, S32, S33, S34 and S35) it was a frequent problem that the ground line was not drawn below the animals. Thus, the leg attitudes relative to the ground were not always clear. This problem was solved in a such a way that in these ambiguous pictures we drew the ground line with the assumption that at least one hind leg and one fore leg is on the ground. In a few cases there were two or three possibilities for the ground line direction. Further details can be gleaned from the Electronic Supplement, where the supposed ground line is displayed in every prehistoric quadruped walking illustration.

### Statistics

For statistical analyses (binomial χ^2^ test) we used Statistica 7.0.

## Supporting Information

Figure S1 **Contour of a cow copied from a picture of a prehistoric painting from Chad.** (The original colour picture can be found in the following website: http://www.bradshawfondation.com). The straight line represents the assumed ground line. LH: left hind leg, LF: left fore leg, RH: right hind leg, RF: right fore leg.
(DOC)

Figure S2 **As Fig. S1 for a prehistoric picture of a horse found near the river Draa (** http://en.wikipedia.org/wiki/Draa_River **).**
(DOC)

Figure S3 **As Fig. S1 for a prehistoric picture of a bull found near the river Draa (** http://en.wikipedia.org/wiki/Draa_River **).**
(DOC)

Figure S4 **As Fig. S1 for a prehistoric picture of a giraffe from Inak (** http://www.bradshawfondation.com **).**
(DOC)

Figure S5 **As Fig. S1 for a prehistoric picture of a horse from the French cave Lascaux (** http://en.wikipedia.org/wiki/Lascaux **).**
(DOC)

Figure S6 **As Fig. S1 for a prehistoric picture of a bull from the French cave Lascaux (** http://pittkyle123.wordpress.com/2011/02/15/cave-paintings-30000-years-ago **).**
(DOC)

Figure S7 **As Fig. S1 for a prehistoric picture of an elephant from the Libian Tadrart Acacus (** http://www.galuzzi.it **).**
(DOC)

Figure S8 **As Fig. S1 for a prehistoric picture of a cow from the French cave Lascaux (** http://www.lascaux.culture.fr **).**
(DOC)

Figure S9 **As Fig. S1 for a prehistoric picture of an antelope from the mountain Drakenberg in Eland (** http://www.superstock.com **).**
(DOC)

Figure S10 **As Fig. S1 for a prehistoric picture of an antelope from the Drakenberg mountain in Eland (** http://www.freewebs.com/maloti/stoneageandbushman.htm **).**
(DOC)

Figure S11 **As Fig. S1 for a prehistoric picture of a bull from the mountain Drakenberg in Eland (** http://www.bradshawfondation.com **).**
(DOC)

Figure S12 **As Fig. S1 for a prehistoric picture of a bull from the mountain Drakenberg in Eland (** http://www-users.york.ac.uk **).**
(DOC)

Figure S13 **As Fig. S1 for a prehistoric picture of a horse from the French cave Lascaux (** http://www.lascaux.culture.fr **).**
(DOC)

Figure S14 **As Fig. S1 for a prehistoric picture of a horse from the French cave Lascaux (** http://www.lascaux.culture.fr **).**
(DOC)

Figure S15 **As Fig. S1 for a prehistoric picture of a bull from the French cave Lascaux (** http://www.lascaux.culture.fr **).**
(DOC)

Figure S16 **As Fig. S1 for a prehistoric picture of a horse from the French cave Lascaux (** http://www.lascaux.culture.fr **).**
(DOC)

Figure S17 **As Fig. S1 for a prehistoric picture of a horse from the French cave Lascaux (** http://www.lascaux.culture.fr **).**
(DOC)

Figure S18 **As Fig. S1 for a prehistoric picture of a horse from the French cave Lascaux (** http://www.lascaux.culture.fr **).**
(DOC)

Figure S19 **As Fig. S1 for a prehistoric picture of a giraffe from the Libian Tadrart Acacus (** http://www.ewpnet.com/libySacacus/index.htm **).**
(DOC)

Figure S20 **As Fig. S1 for a prehistoric picture of a deer from the Spanish cave Altamira (** http://popular-archaeology.com/issue/september-2011/article/saving-altamira-cave **).**
(DOC)

Figure S21 **As Fig. S1 for a prehistoric picture of an elephant from the Libian Tadrart Acacus (** http://www.willgoto.com **).**
(DOC)

Figure S22 **As Fig. S1 for a prehistoric picture of a giraffe from the Libian Tadrart Acacus (** http://www.flickr.com **).**
(DOC)

Figure S23 **As Fig. S1 for a prehistoric picture of a buffalo from the Libian Tadrart Acacus (** http://www.arcl.ed.ac.uk **).**
(DOC)

Figure S24 **As Fig. S1 for a prehistoric picture of a rhinoceros from the French cave Niaux (** http://www.bradshawfondation.com **).**
(DOC)

Figure S25 **As Fig. S1 for a prehistoric picture of a deer from the Indian Shamla Hill (** http://www.bradshawfondation.com **).**
(DOC)

Figure S26 **As Fig. S1 for a prehistoric picture of a cow from India (** http://www.bradshawfondation.com **).**
(DOC)

Figure S27 **As Fig. S1 for a prehistoric picture of a bull from India (** http://www.bradshawfondation.com **).**
(DOC)

Figure S28 **As Fig. S1 for a prehistoric picture of an antelope from India (** http://www.bradshawfondation.com **).**
(DOC)

Figure S29 **As Fig. S1 for a prehistoric picture of an antelope from India (** http://www.bradshawfondation.com **).**
(DOC)

Figure S30 **As Fig. S1 for a prehistoric picture of an antelope from India (** http://www.bradshawfondation.com **).**
(DOC)

Figure S31 **As Fig. S1 for a prehistoric picture of a deer from India (** http://www.bradshawfondation.com **).**
(DOC)

Figure S32 **As Fig. S1 for a prehistoric picture of a quadruped from the Indian Bhimabetaka (** http://bmaks.webs.com/cavepaintings.htm **).**
(DOC)

Figure S33 **As Fig. S1 for a prehistoric picture of a bull from India (** http://whc.unesco.org/en/list/925 **).**
(DOC)

Figure S34 **As Fig. S1 for a prehistoric picture of a mammoth from the Indian Karabad (** http://www.bradshawfondation.com **).**
(DOC)

Figure S35 **As Fig. S1 for a prehistoric picture of an elephant from the Indian Bhimabetaka (** http://bmaks.webs.com/cavepaintings.htm **).**
(DOC)

Table S1 The numbers of **all modern** (after prehistory) correct (grey cells) and incorrect (white cells) quadruped walking illustrations in the walking matrix. *N*_correct_ = 334, *N*_incorrect_ = 627, total *N* = *N*_correct_+*N*_incorrect_ = 961. The error rate is *r* = *N*_incorrect_/*N* = 65.2%.
(DOC)

Table S2 The numbers of correct (grey cells) and incorrect (white cells) **prehistoric** quadruped walking illustrations (see Supporting Information S1) in the walking matrix. *N*_correct_ = 21, *N*_incorrect_ = 18, total *N* = *N*_correct_+*N*_incorrect_ = 39. The error rate is *r* = *N*_incorrect_/*N* = 46.2%.
(DOC)

Table S3 The numbers of **all (prehistoric and modern)** correct (grey cells) and incorrect (white cells) quadruped walking illustrations in the walking matrix. *N*_correct_ = 355, *N*_incorrect_ = 645, total *N* = *N*_correct_+*N*_incorrect_ = 1000. The error rate is *r* = *N*_incorrect_/*N* = 64.5%.
(DOC)

Table S4 The numbers of correct (grey cells) and incorrect (white cells) **pre-Muybridgean** (after prehistory and prior to 1887) quadruped walking illustrations in the walking matrix. *N*_correct_ = 45, *N*_incorrect_ = 227, total *N* = *N*_correct_+*N*_incorrect_ = 272. The error rate is *r* = *N*_incorrect_/*N* = 83.5%.
(DOC)

Table S5 The numbers of correct (grey cells) and incorrect (white cells) **post-Muybridgean** (after 1887) quadruped walking illustrations in the walking matrix. *N*_correct_ = 289, *N*_incorrect_ = 397, total *N* = *N*_correct_+*N*_incorrect_ = 686. The error rate is *r* = *N*_incorrect_/*N* = 57.9%.
(DOC)

Table S6 The numbers of correct (grey cells) and incorrect (white cells) **cavalry statues** in the walking matrix. *N*_correct_ = 124, *N*_incorrect_ = 235, total *N* = *N*_correct_+*N*_incorrect_ = 359. The error rate is *r* = *N*_incorrect_/*N* = 65.5%.
(DOC)

Table S7 The numbers of correct (grey cells) and incorrect (white cells) **two-dimensional (paintings, graphic art, reliefs)** quadruped walking illustrations in the walking matrix. *N*_correct_ = 210, *N*_incorrect_ = 392, total *N* = *N*_correct_+*N*_incorrect_ = 602. The error rate is *r* = *N*_incorrect_/*N* = 65.1%.
(DOC)

Table S8 The numbers of correct (grey cells) and incorrect (white cells) **horse walking illustrations** in the walking matrix. *N*_correct_ = 244, *N*_incorrect_ = 585, total *N* = *N*_correct_+*N*_incorrect_ = 829. The error rate is *r* = *N*_incorrect_/*N* = 70.6%.
(DOC)

Table S9 The unity walking matrix with number 1 in its every cell. In this case the numbers of correct (grey cells) and incorrect (white cells) quadruped walking illustrations are: *N*_correct_ = 16, *N*_incorrect_ = 44, total *N* = *N*_correct_+*N*_incorrect_ = 60. Then the error rate is *r* = *N*_incorrect_/*N* = 73.3% corresponding with the pure accident.
(DOC)

Supporting Information S1 **Detailed analysis of prehistoric quadruped walking illustrations.**
(DOC)

Supporting Information S2 **Permission for the use of the colour picture in** **Figure 2** **.**
(DOC)
