# Supplementary figures and images for: Cavemen Were Better at Depicting Quadruped Walking than Modern Artists: Erroneous Walking Illustrations in the Fine Arts from Prehistory to Today
Source: PLoS One. 2012 Dec 5;7(12):e49786. doi: 10.1371/journal.pone.0049786 (PMC3515592; doi:10.1371/journal.pone.0049786)

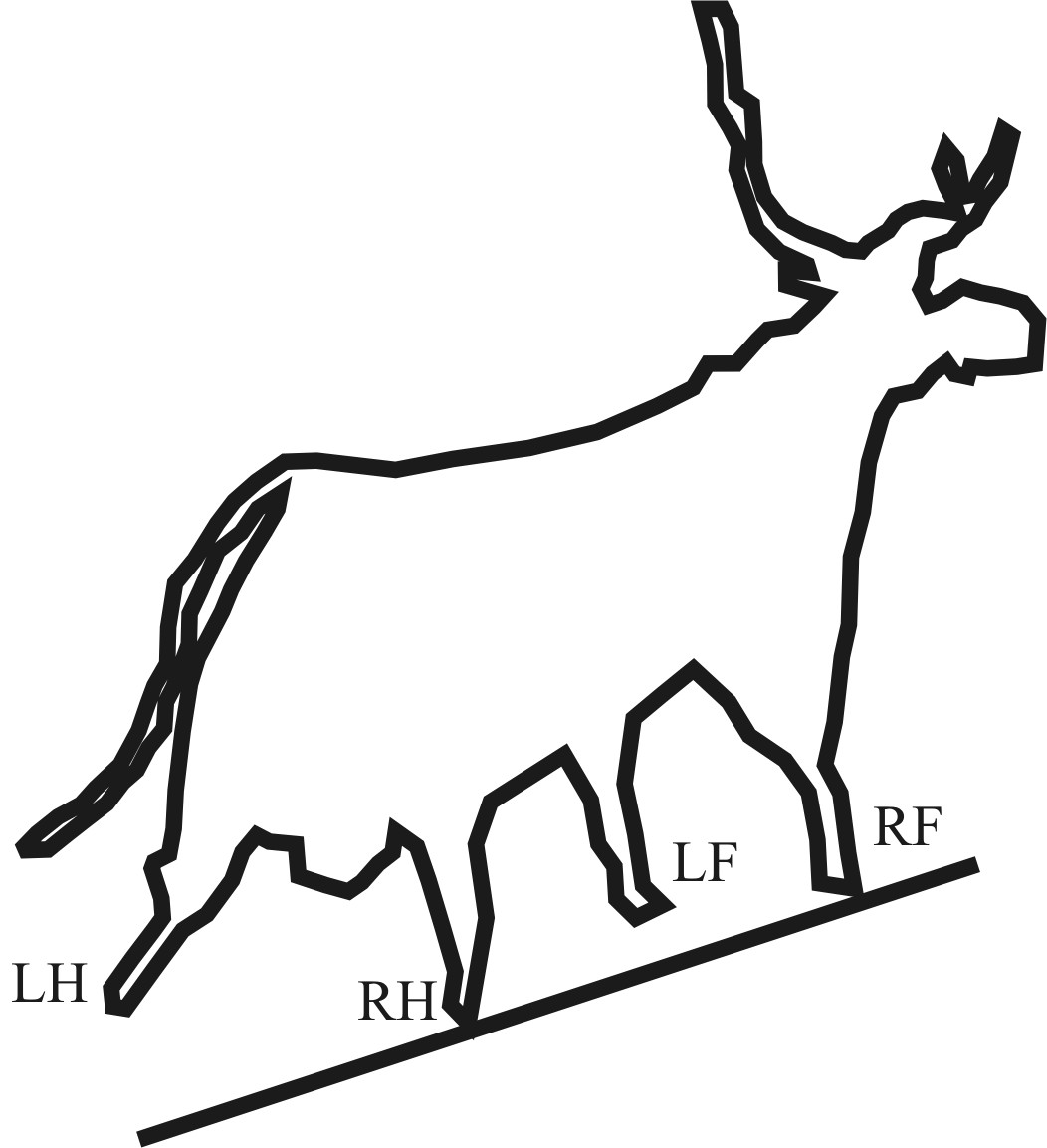


**Supplementary Figure S1**

Supplement: Figure S1 — Contour of a cow copied from a picture of a prehistoric painting from Chad. (The original colour picture can be found in the following website: http://www.bradshawfondation.com). The straight line represents the assumed ground line. LH: left hind leg, LF: left fore leg, RH: right hind leg, RF: right fore leg. (DOC) [file pone.0049786.s001.doc]

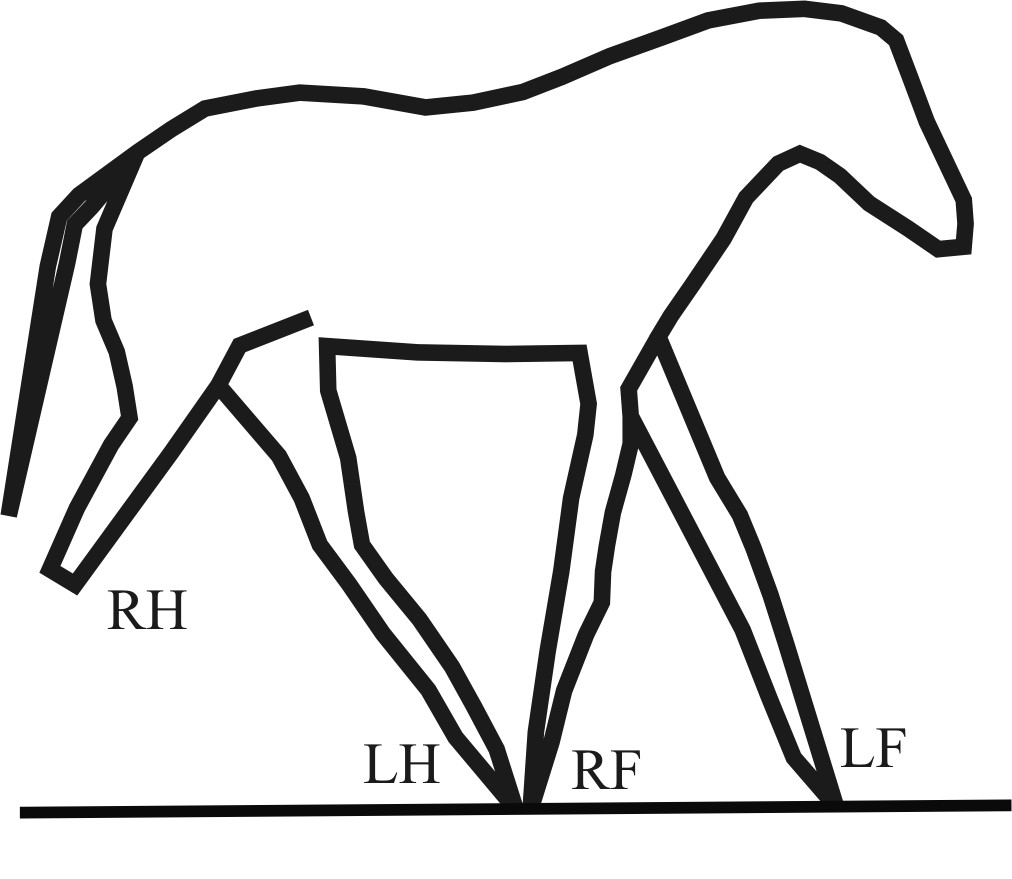


**Supplementary Figure S2**

Supplement: Figure S2 — As Fig. S1 for a prehistoric picture of a horse found near the river Draa ( http://en.wikipedia.org/wiki/Draa_River ). (DOC) [file pone.0049786.s002.doc]

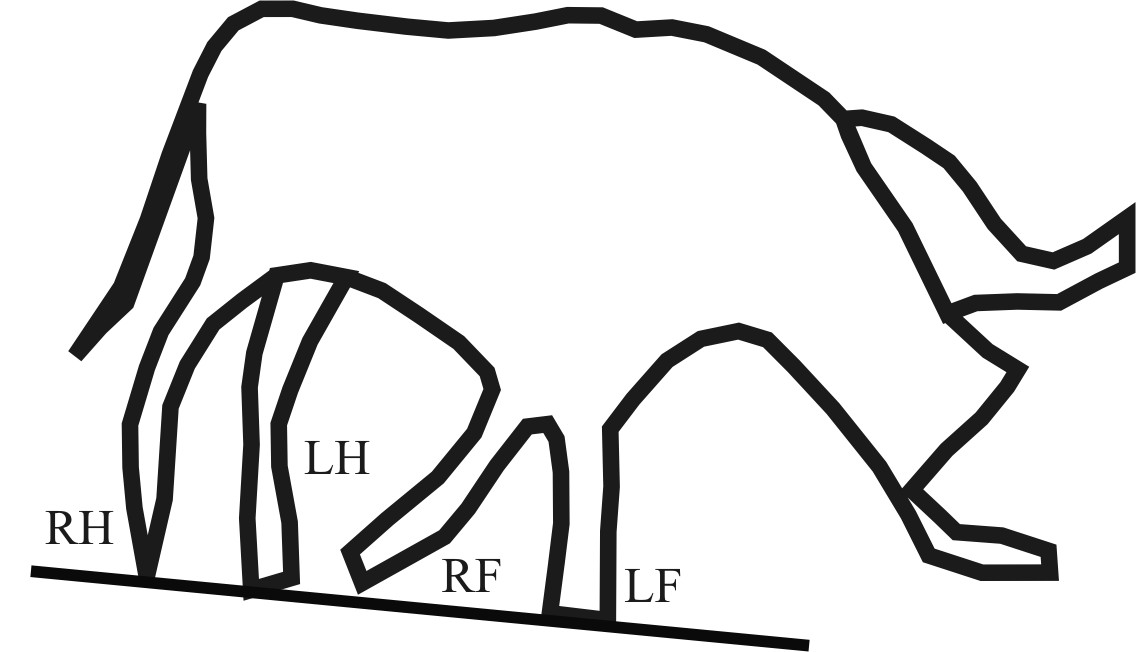


**Supplementary Figure S3**

Supplement: Figure S3 — As Fig. S1 for a prehistoric picture of a bull found near the river Draa ( http://en.wikipedia.org/wiki/Draa_River ). (DOC) [file pone.0049786.s003.doc]

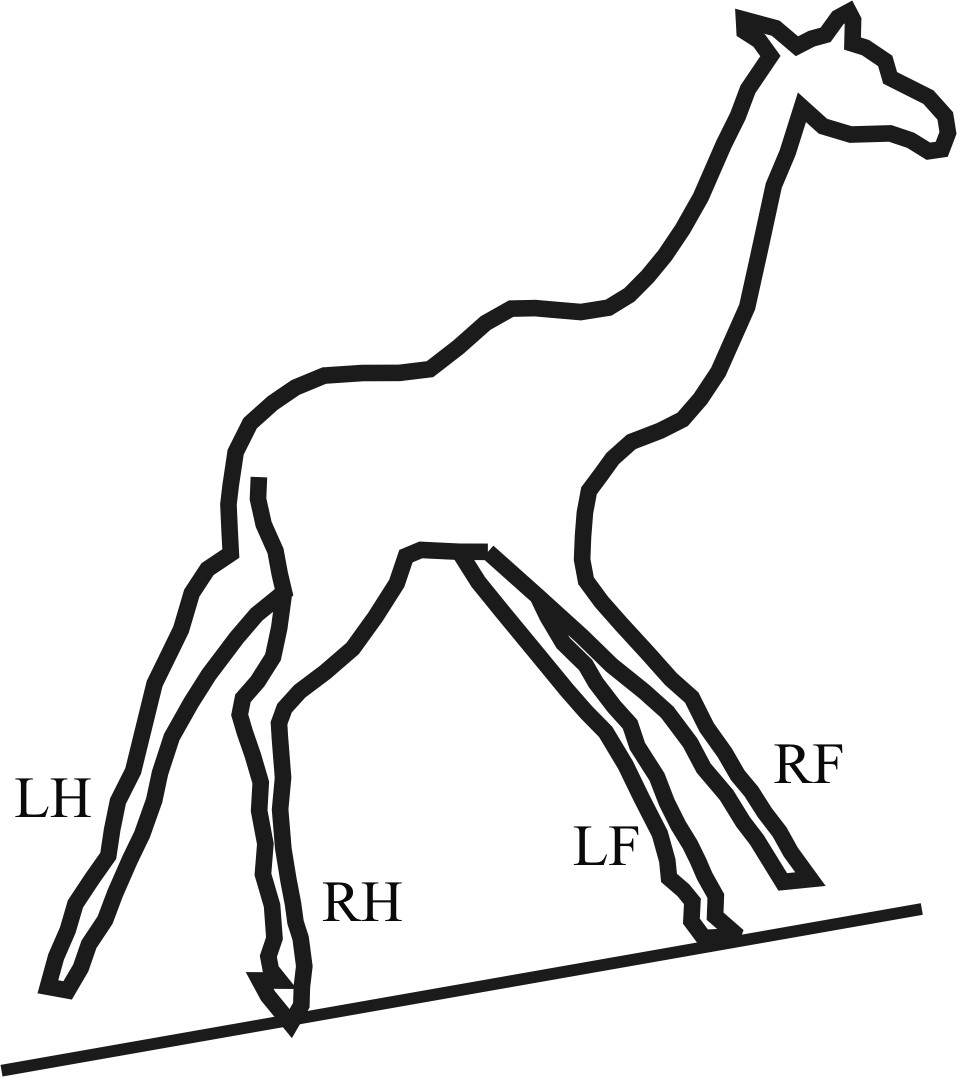


**Supplementary Figure S4**

Supplement: Figure S4 — As Fig. S1 for a prehistoric picture of a giraffe from Inak ( http://www.bradshawfondation.com ). (DOC) [file pone.0049786.s004.doc]

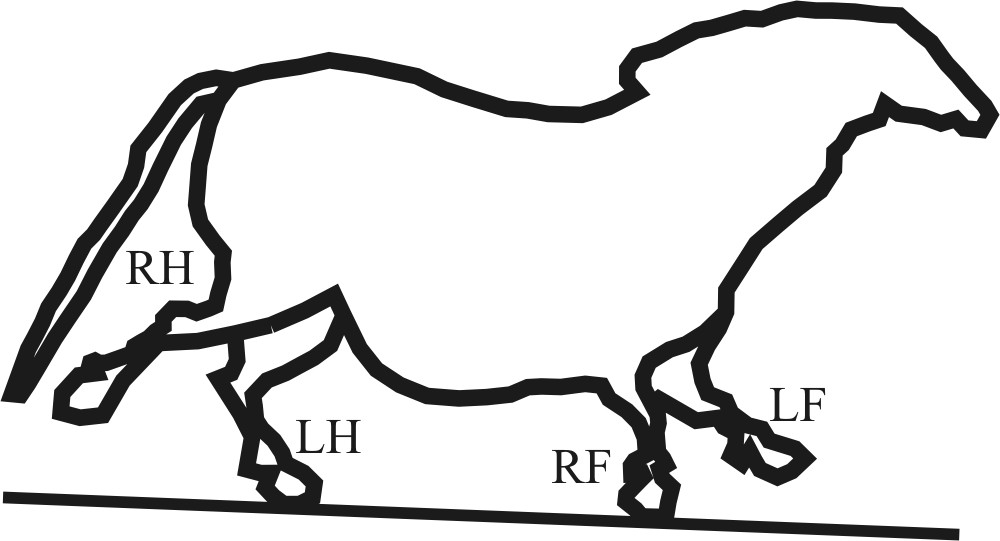


**Supplementary Figure S5**

Supplement: Figure S5 — As Fig. S1 for a prehistoric picture of a horse from the French cave Lascaux ( http://en.wikipedia.org/wiki/Lascaux ). (DOC) [file pone.0049786.s005.doc]

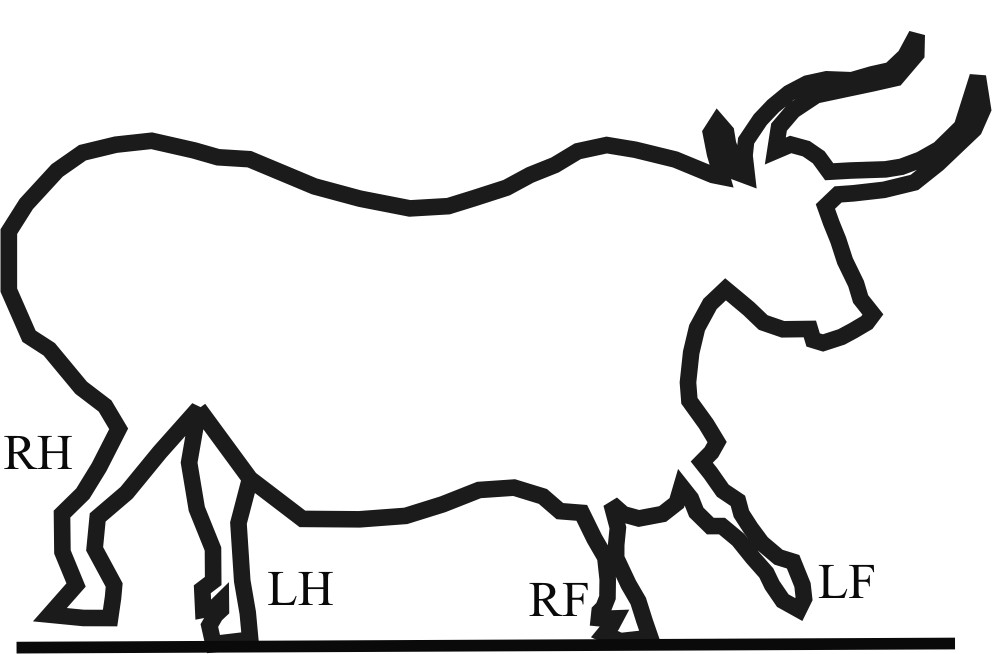


**Supplementary Figure S6**

Supplement: Figure S6 — As Fig. S1 for a prehistoric picture of a bull from the French cave Lascaux ( http://pittkyle123.wordpress.com/2011/02/15/cave-paintings-30000-years-ago ). (DOC) [file pone.0049786.s006.doc]

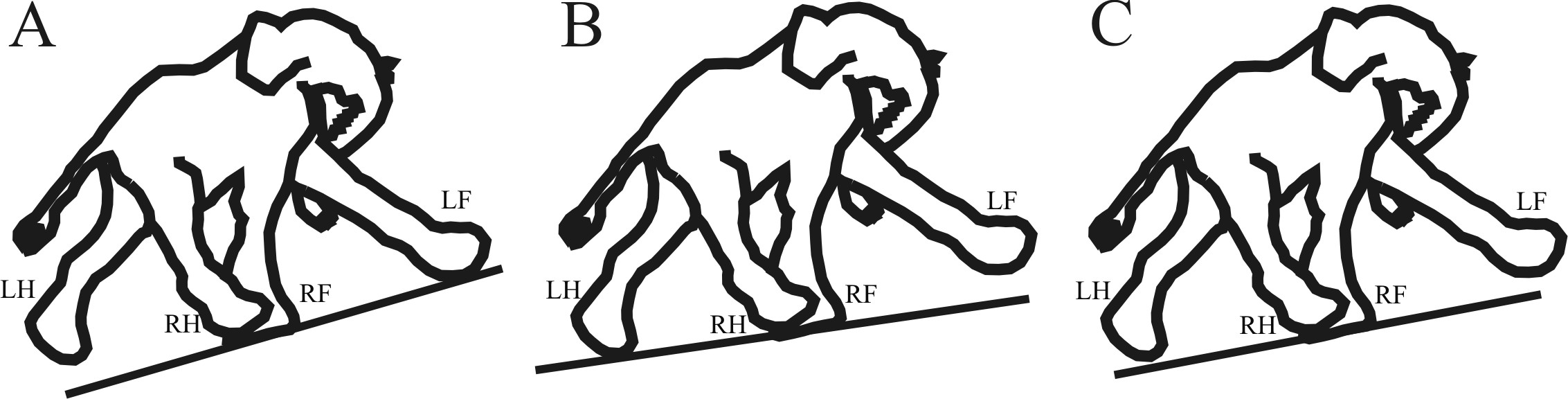


**Supplementary Figure S7**

Supplement: Figure S7 — As Fig. S1 for a prehistoric picture of an elephant from the Libian Tadrart Acacus ( http://www.galuzzi.it ). (DOC) [file pone.0049786.s007.doc]

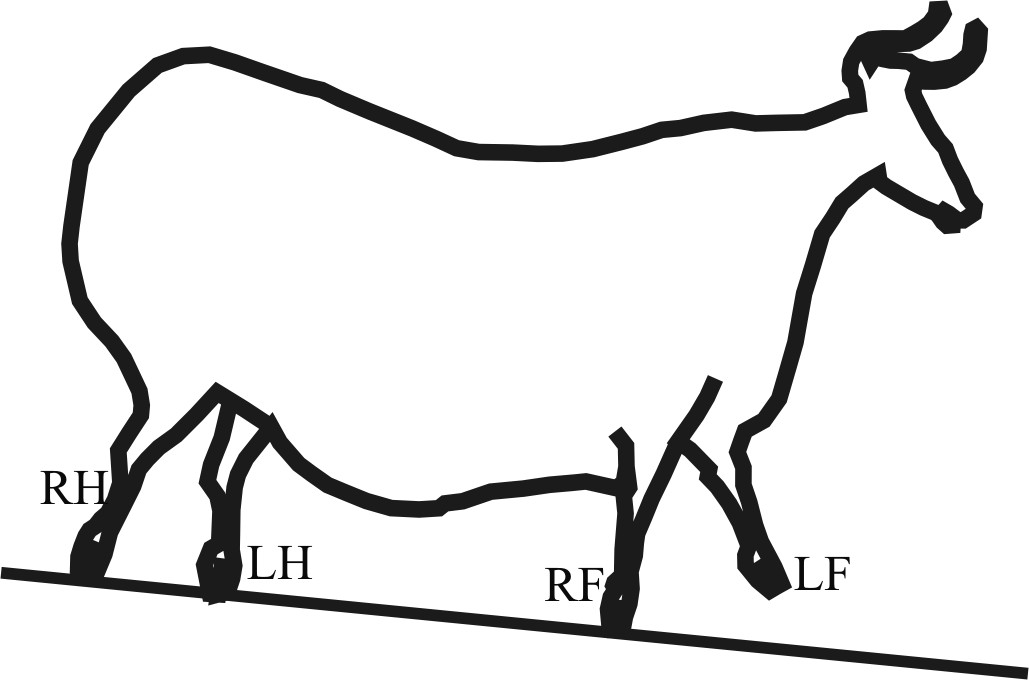


**Supplementary Figure S8**

Supplement: Figure S8 — As Fig. S1 for a prehistoric picture of a cow from the French cave Lascaux ( http://www.lascaux.culture.fr ). (DOC) [file pone.0049786.s008.doc]

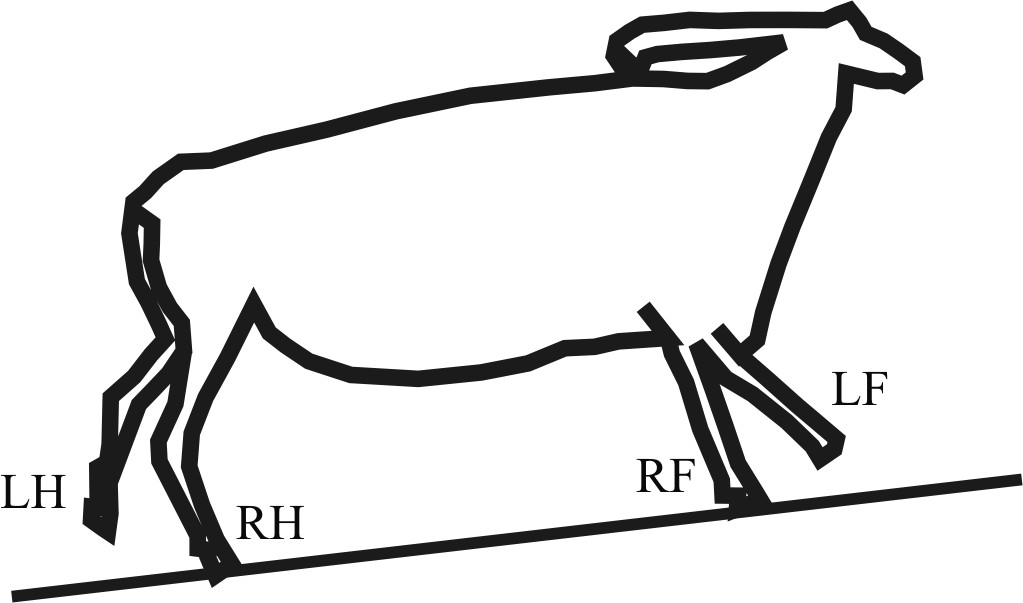


**Supplementary Figure S9**

Supplement: Figure S9 — As Fig. S1 for a prehistoric picture of an antelope from the mountain Drakenberg in Eland ( http://www.superstock.com ). (DOC) [file pone.0049786.s009.doc]

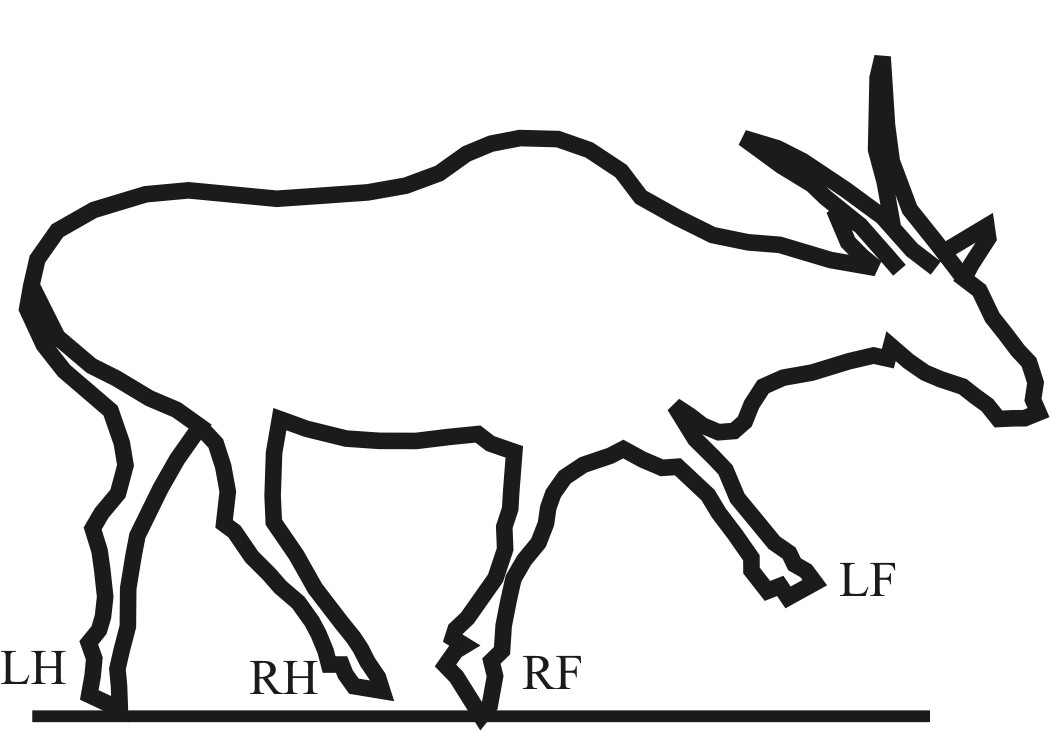


**Supplementary Figure S10**

Supplement: Figure S10 — As Fig. S1 for a prehistoric picture of an antelope from the Drakenberg mountain in Eland ( http://www.freewebs.com/maloti/stoneageandbushman.htm ). (DOC) [file pone.0049786.s010.doc]

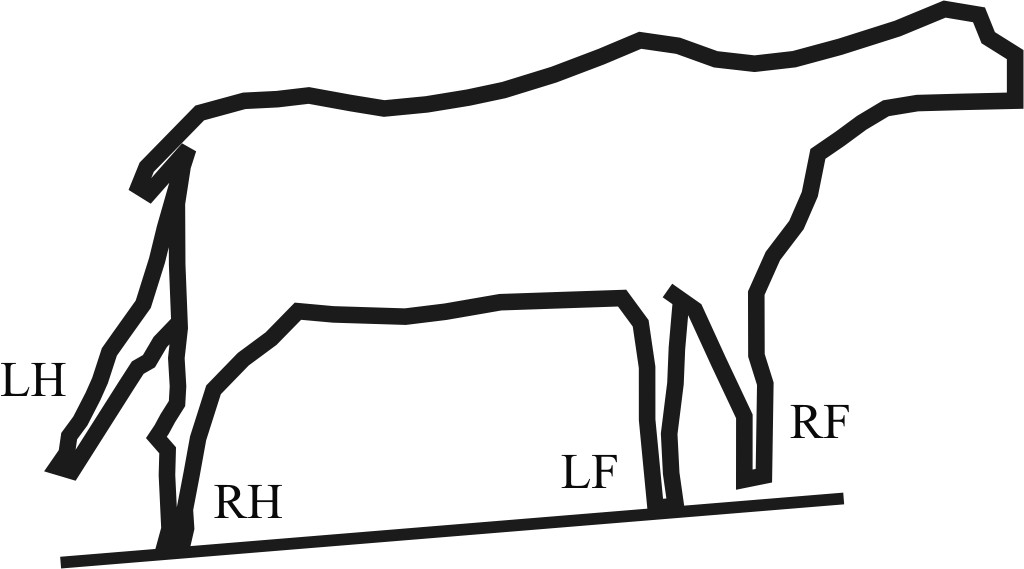


**Supplementary Figure S11**

Supplement: Figure S11 — As Fig. S1 for a prehistoric picture of a bull from the mountain Drakenberg in Eland ( http://www.bradshawfondation.com ). (DOC) [file pone.0049786.s011.doc]

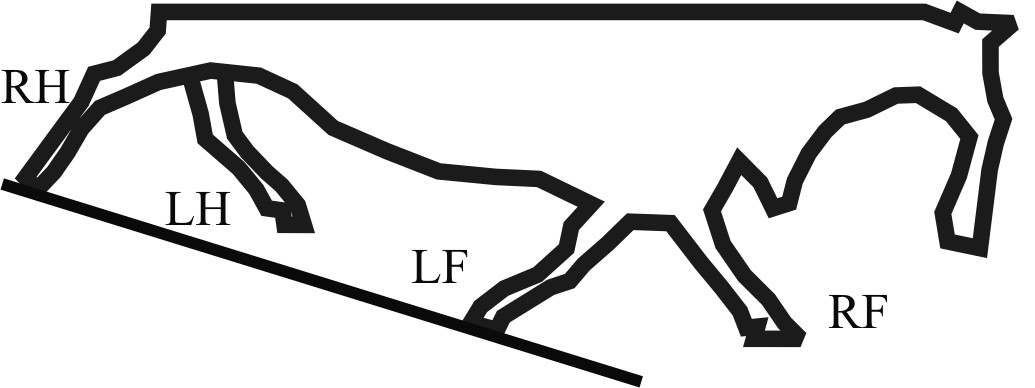


**Supplementary Figure S12**

Supplement: Figure S12 — As Fig. S1 for a prehistoric picture of a bull from the mountain Drakenberg in Eland ( http://www-users.york.ac.uk ). (DOC) [file pone.0049786.s012.doc]

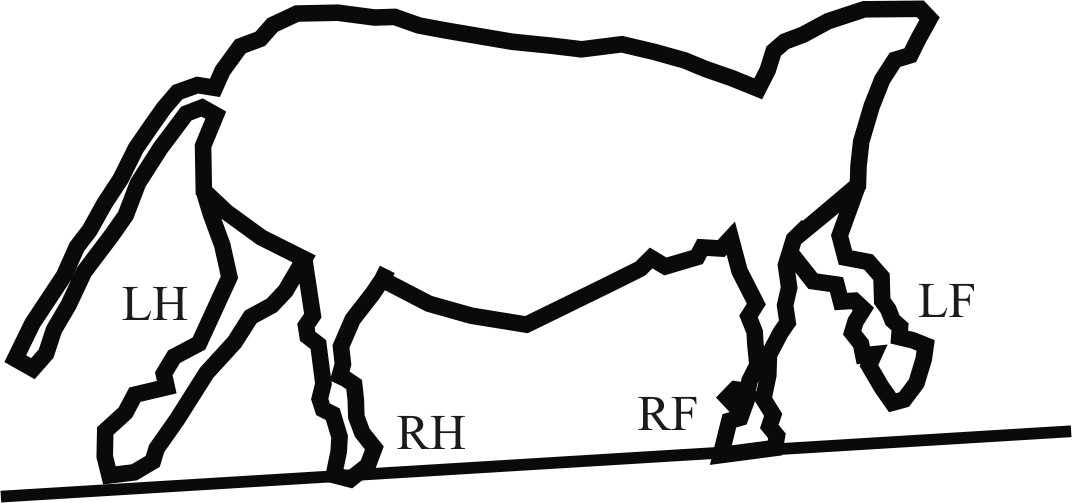


**Supplementary Figure S13**

Supplement: Figure S13 — As Fig. S1 for a prehistoric picture of a horse from the French cave Lascaux ( http://www.lascaux.culture.fr ). (DOC) [file pone.0049786.s013.doc]

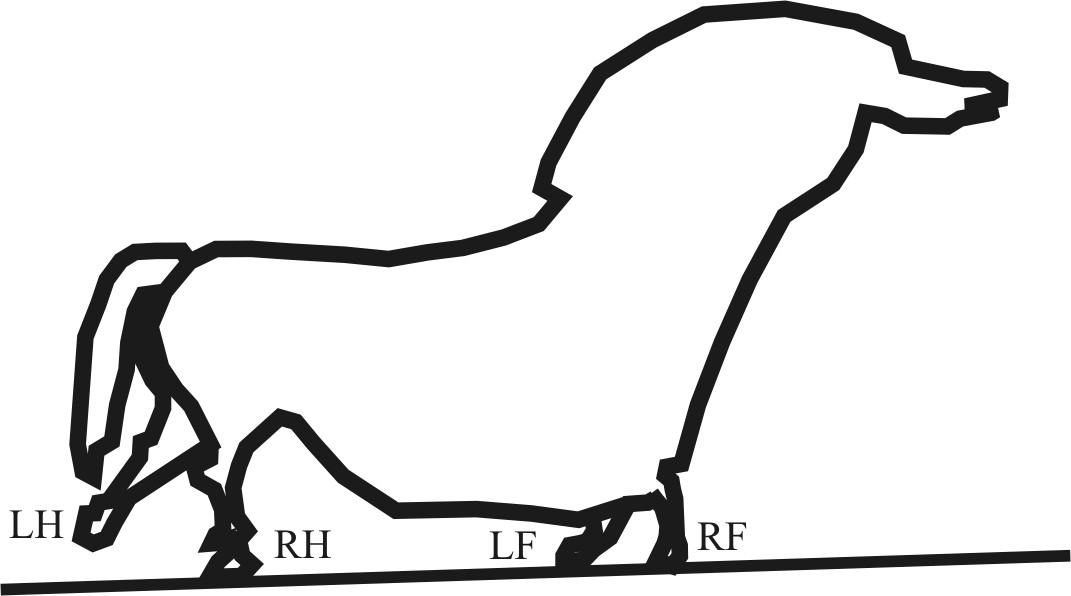


**Supplementary Figure S14**

Supplement: Figure S14 — As Fig. S1 for a prehistoric picture of a horse from the French cave Lascaux ( http://www.lascaux.culture.fr ). (DOC) [file pone.0049786.s014.doc]

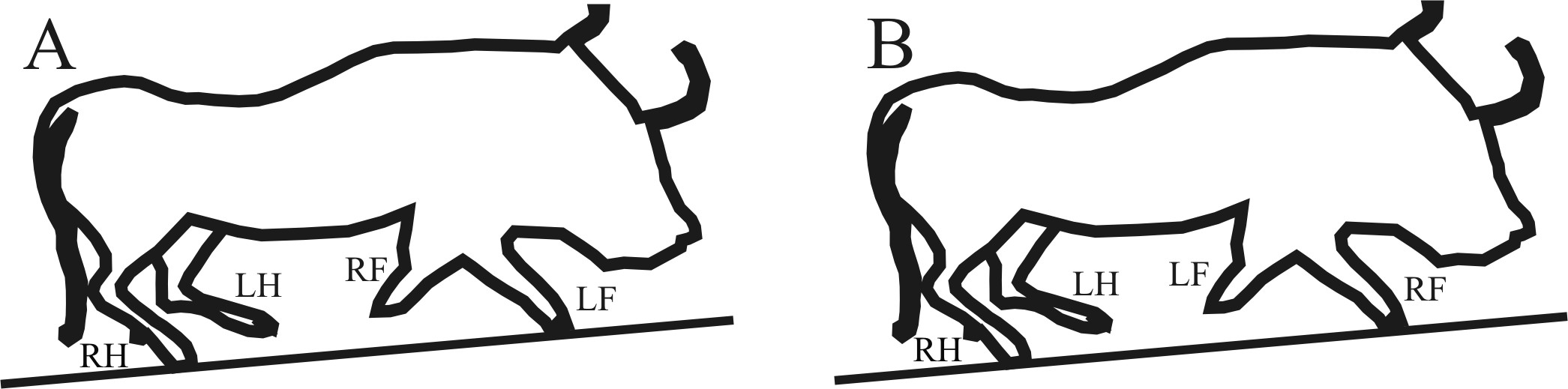


**Supplementary Figure S15**

Supplement: Figure S15 — As Fig. S1 for a prehistoric picture of a bull from the French cave Lascaux ( http://www.lascaux.culture.fr ). (DOC) [file pone.0049786.s015.doc]

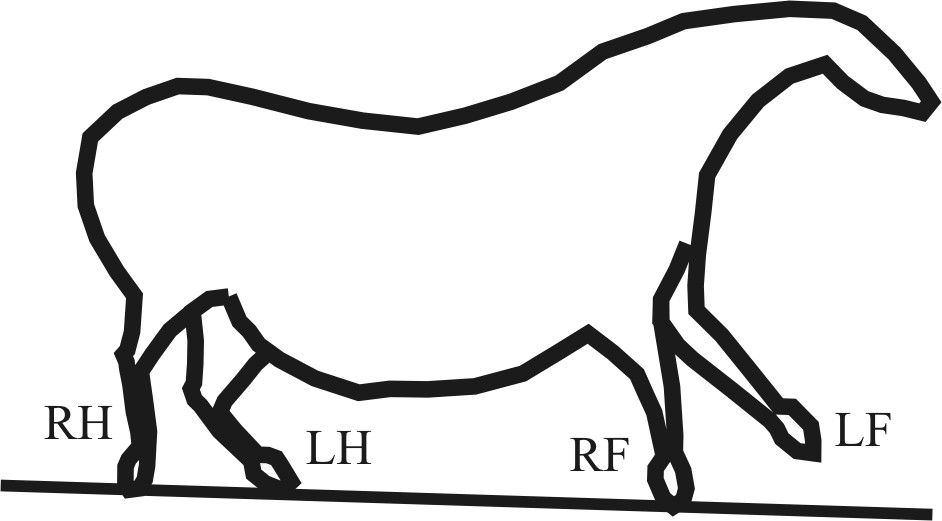


**Supplementary Figure S16**

Supplement: Figure S16 — As Fig. S1 for a prehistoric picture of a horse from the French cave Lascaux ( http://www.lascaux.culture.fr ). (DOC) [file pone.0049786.s016.doc]

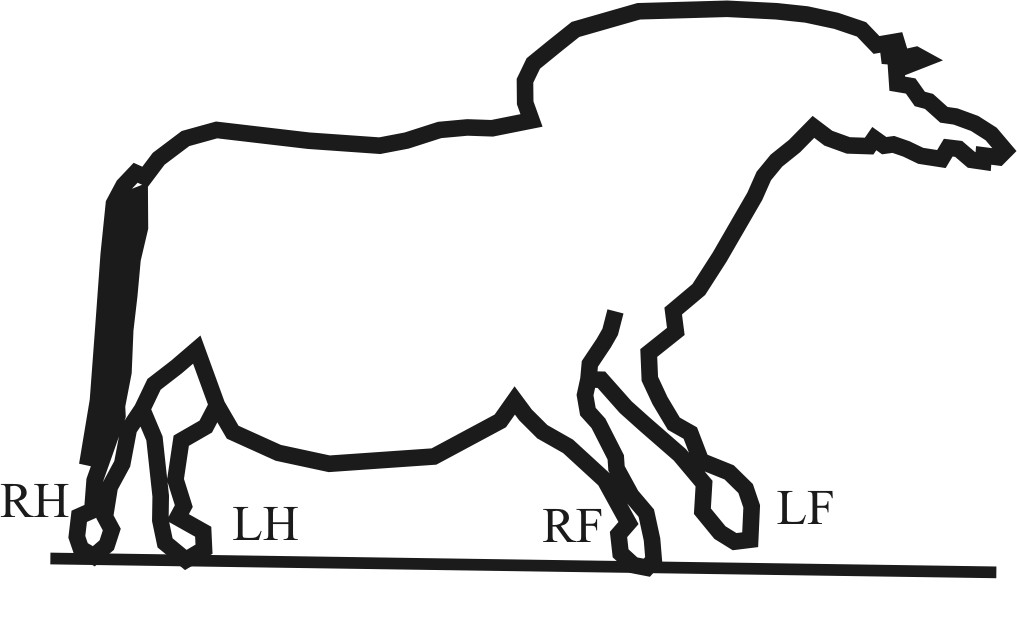


**Supplementary Figure S17**

Supplement: Figure S17 — As Fig. S1 for a prehistoric picture of a horse from the French cave Lascaux ( http://www.lascaux.culture.fr ). (DOC) [file pone.0049786.s017.doc]

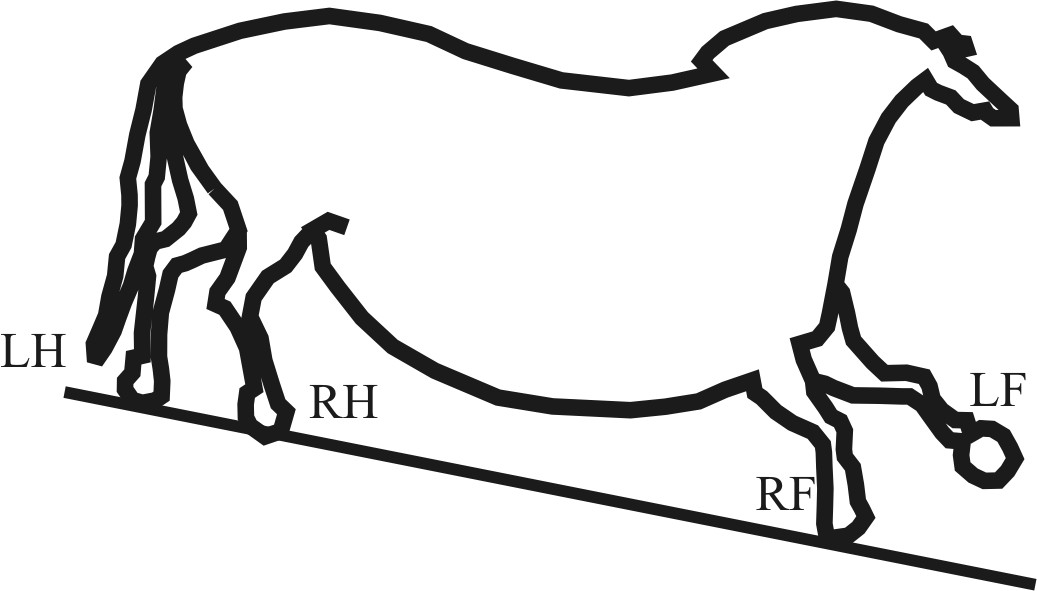


**Supplementary Figure S18**

Supplement: Figure S18 — As Fig. S1 for a prehistoric picture of a horse from the French cave Lascaux ( http://www.lascaux.culture.fr ). (DOC) [file pone.0049786.s018.doc]

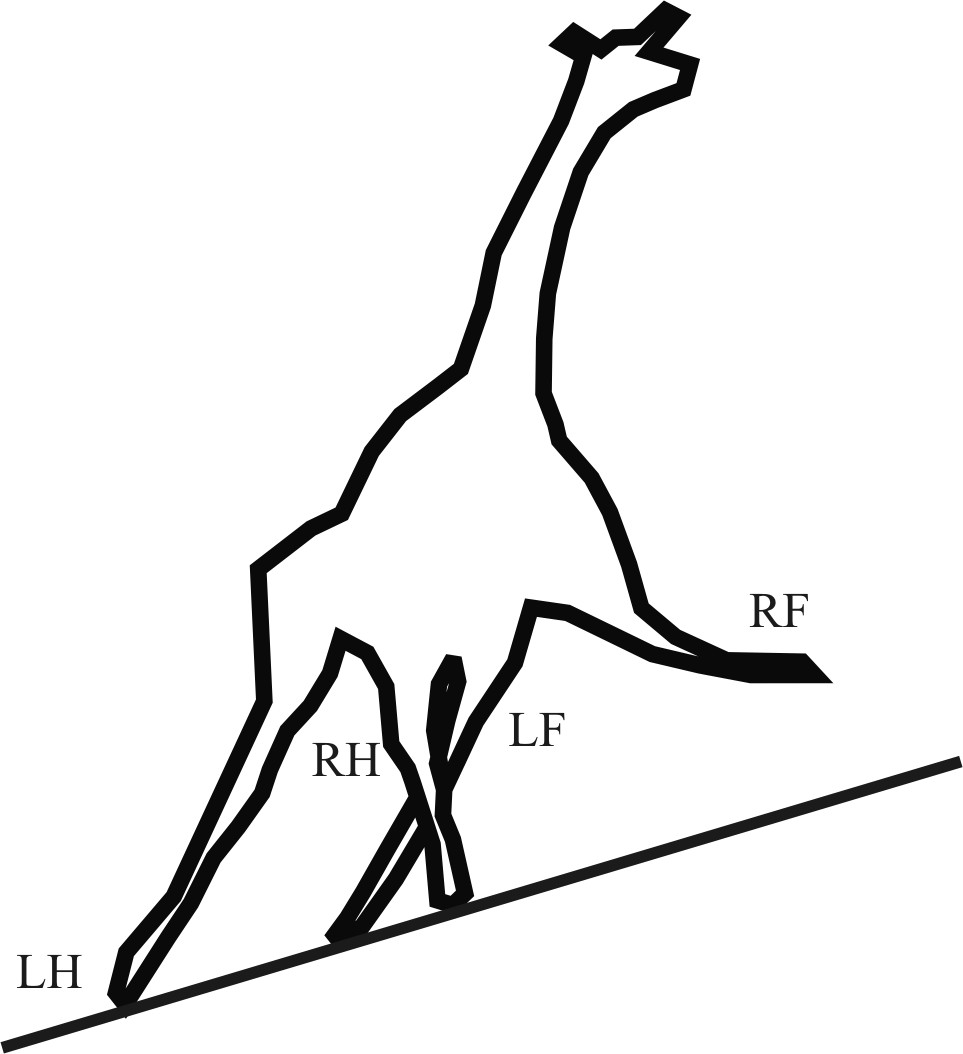


**Supplementary Figure S19**

Supplement: Figure S19 — As Fig. S1 for a prehistoric picture of a giraffe from the Libian Tadrart Acacus ( http://www.ewpnet.com/libySacacus/index.htm ). (DOC) [file pone.0049786.s019.doc]

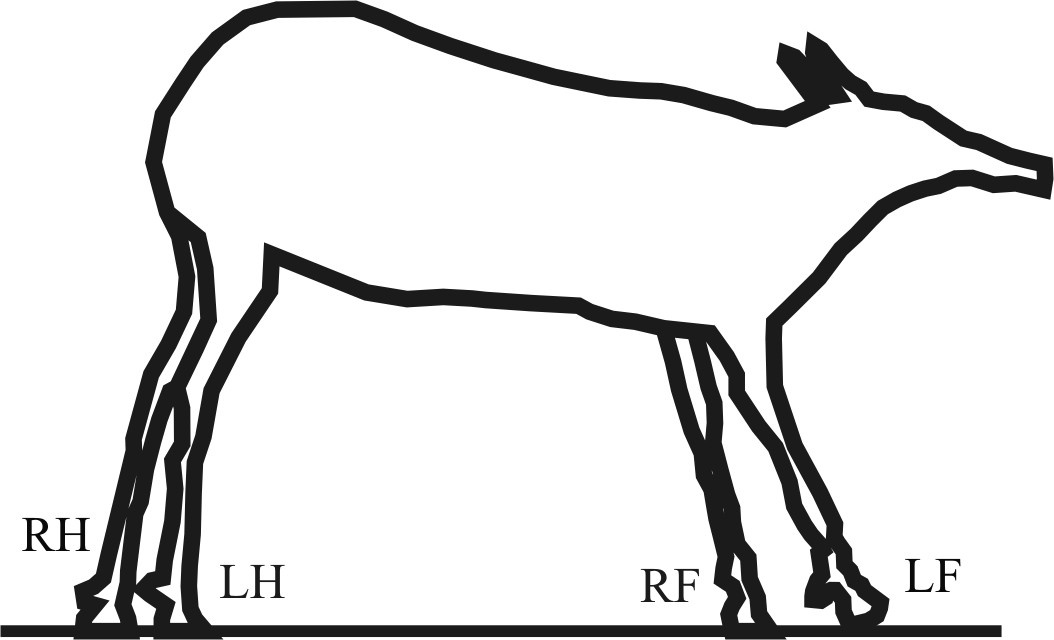


**Supplementary Figure S20**

Supplement: Figure S20 — As Fig. S1 for a prehistoric picture of a deer from the Spanish cave Altamira ( http://popular-archaeology.com/issue/september-2011/article/saving-altamira-cave ). (DOC) [file pone.0049786.s020.doc]

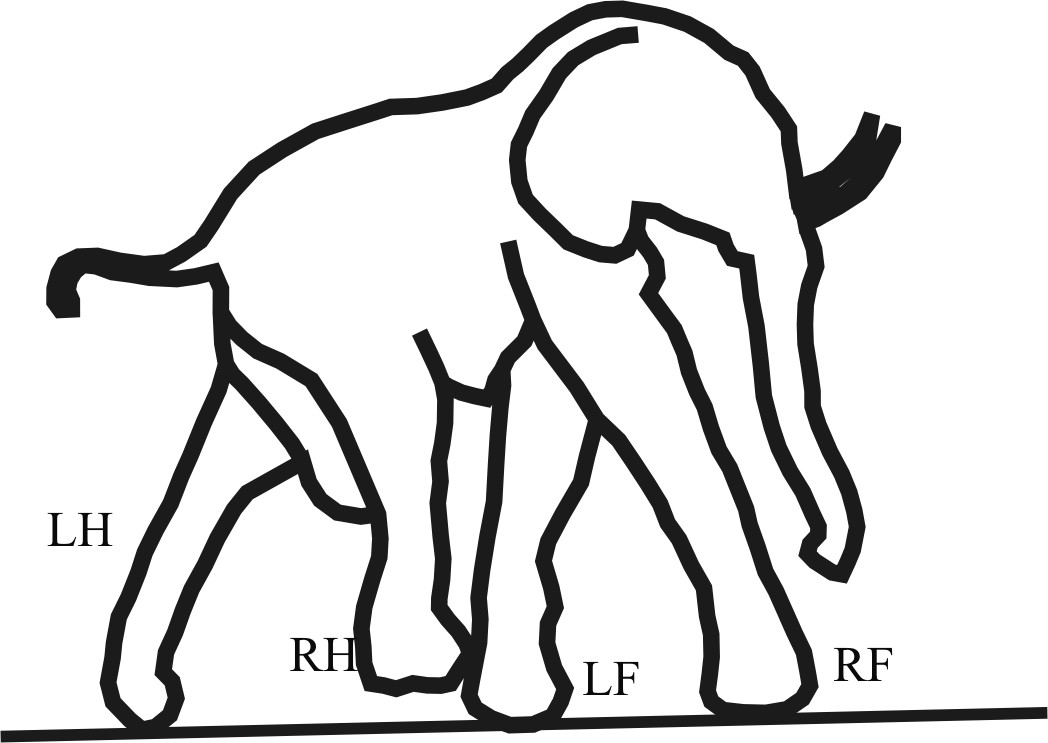


**Supplementary Figure S21**

Supplement: Figure S21 — As Fig. S1 for a prehistoric picture of an elephant from the Libian Tadrart Acacus ( http://www.willgoto.com ). (DOC) [file pone.0049786.s021.doc]

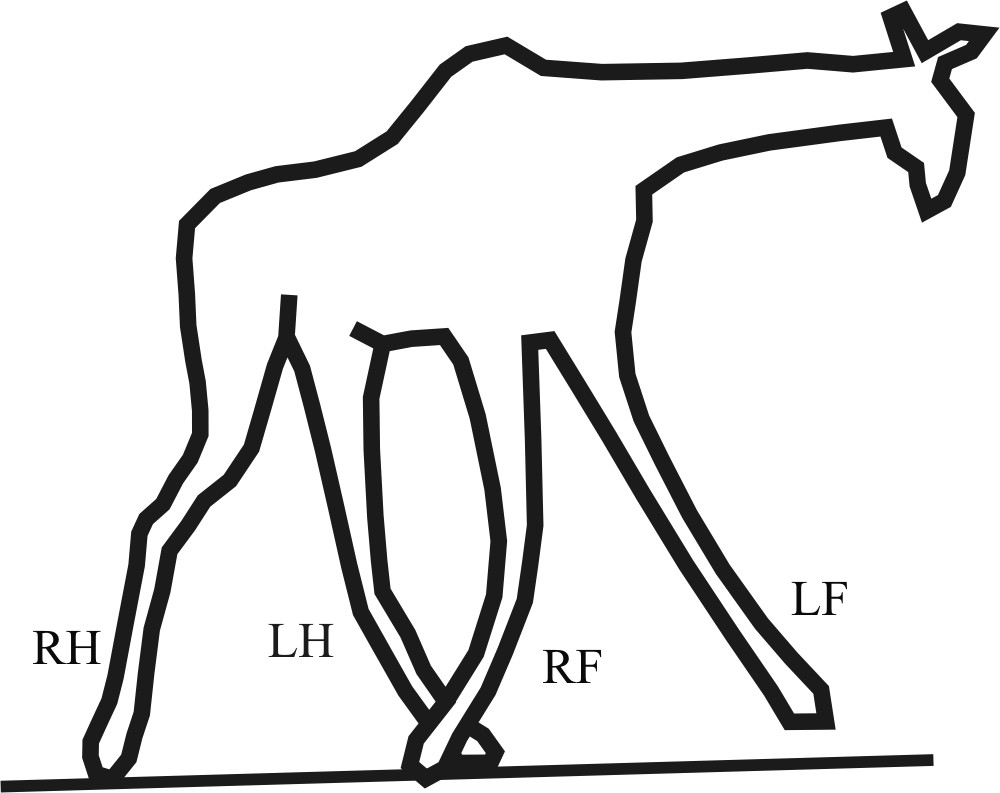


**Supplementary Figure S22**

Supplement: Figure S22 — As Fig. S1 for a prehistoric picture of a giraffe from the Libian Tadrart Acacus ( http://www.flickr.com ). (DOC) [file pone.0049786.s022.doc]

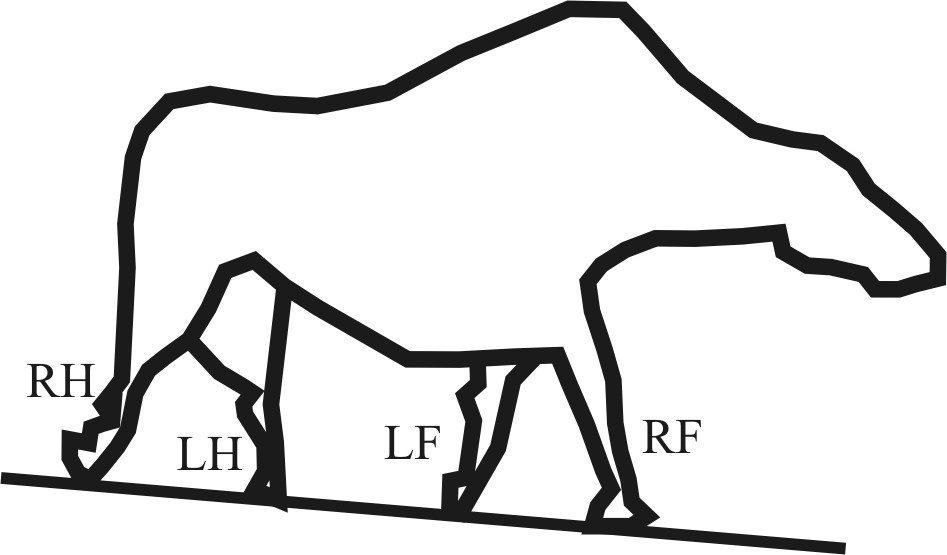


**Supplementary Figure S23**

Supplement: Figure S23 — As Fig. S1 for a prehistoric picture of a buffalo from the Libian Tadrart Acacus ( http://www.arcl.ed.ac.uk ). (DOC) [file pone.0049786.s023.doc]

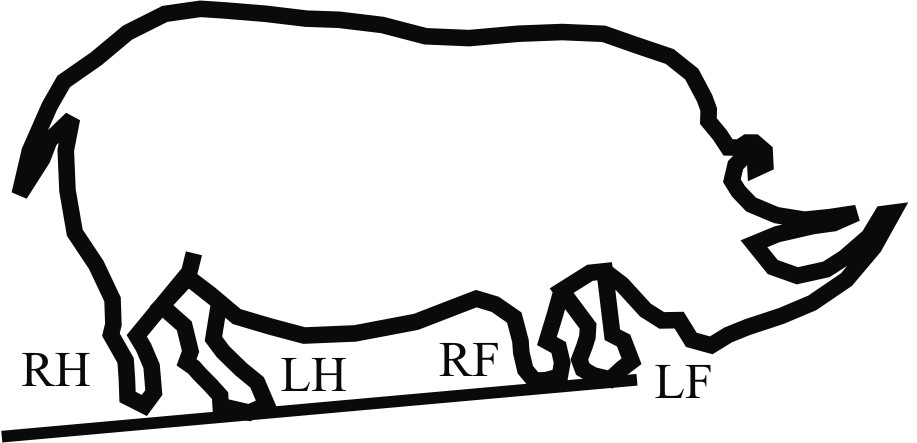


**Supplementary Figure S24**

Supplement: Figure S24 — As Fig. S1 for a prehistoric picture of a rhinoceros from the French cave Niaux ( http://www.bradshawfondation.com ). (DOC) [file pone.0049786.s024.doc]

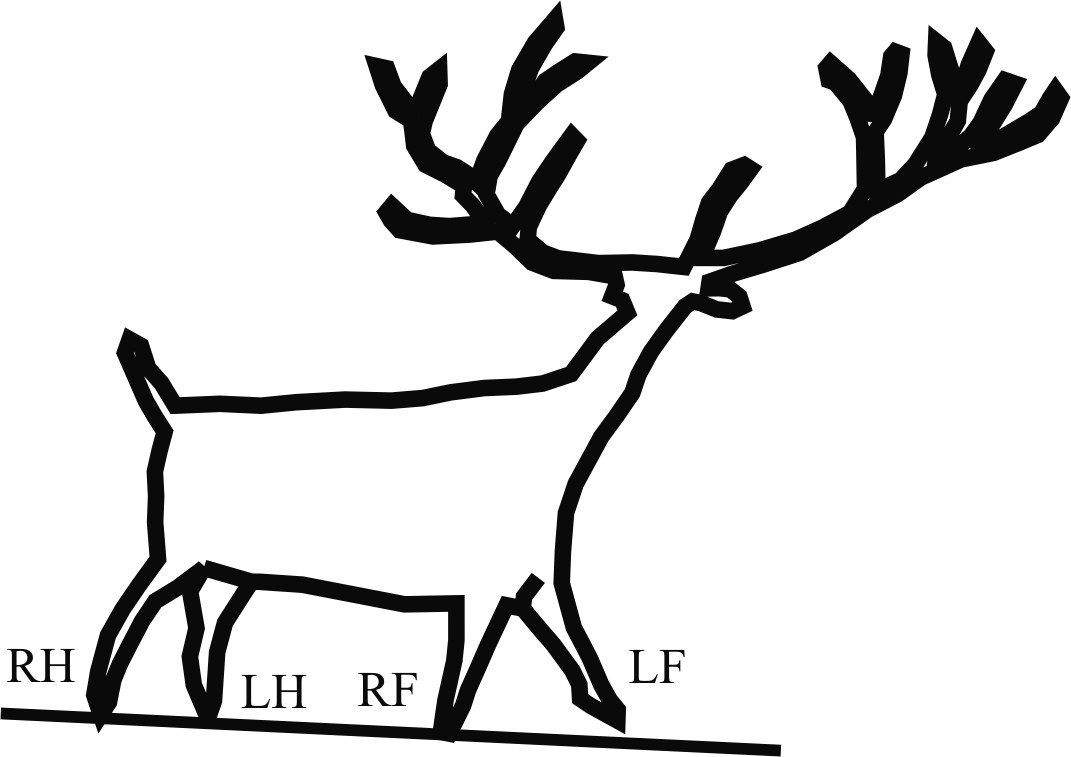


**Supplementary Figure S25**

Supplement: Figure S25 — As Fig. S1 for a prehistoric picture of a deer from the Indian Shamla Hill ( http://www.bradshawfondation.com ). (DOC) [file pone.0049786.s025.doc]

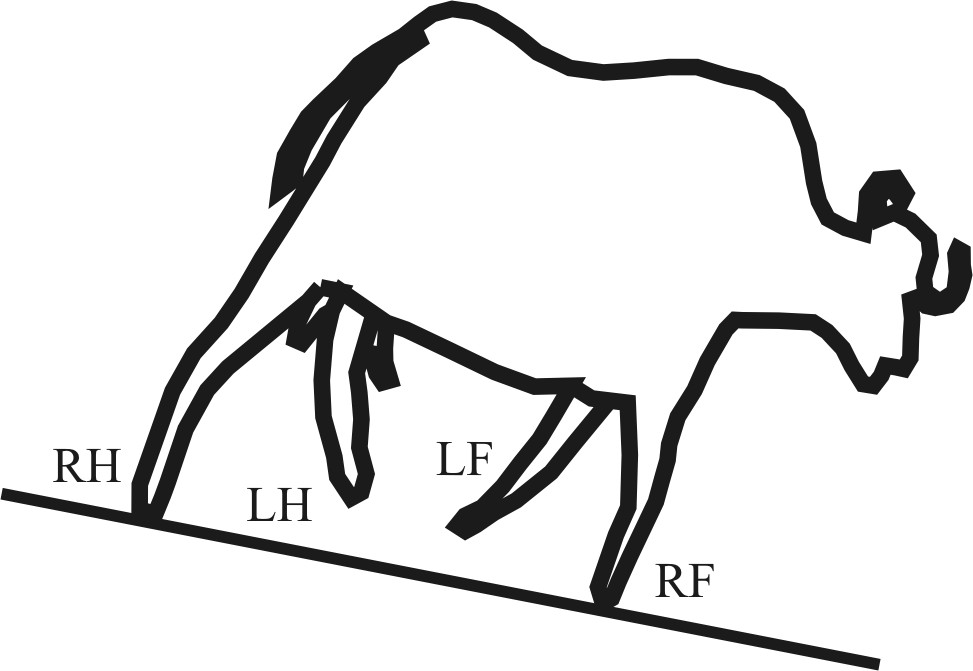


**Supplementary Figure S26**

Supplement: Figure S26 — As Fig. S1 for a prehistoric picture of a cow from India ( http://www.bradshawfondation.com ). (DOC) [file pone.0049786.s026.doc]

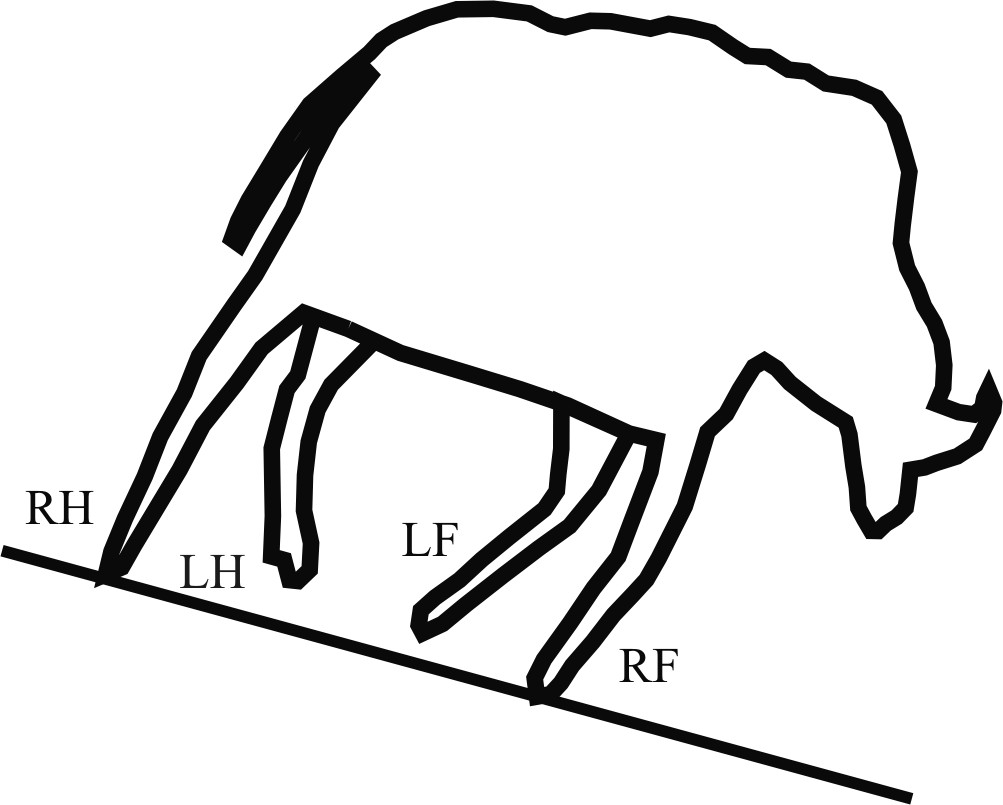


**Supplementary Figure S27**

Supplement: Figure S27 — As Fig. S1 for a prehistoric picture of a bull from India ( http://www.bradshawfondation.com ). (DOC) [file pone.0049786.s027.doc]

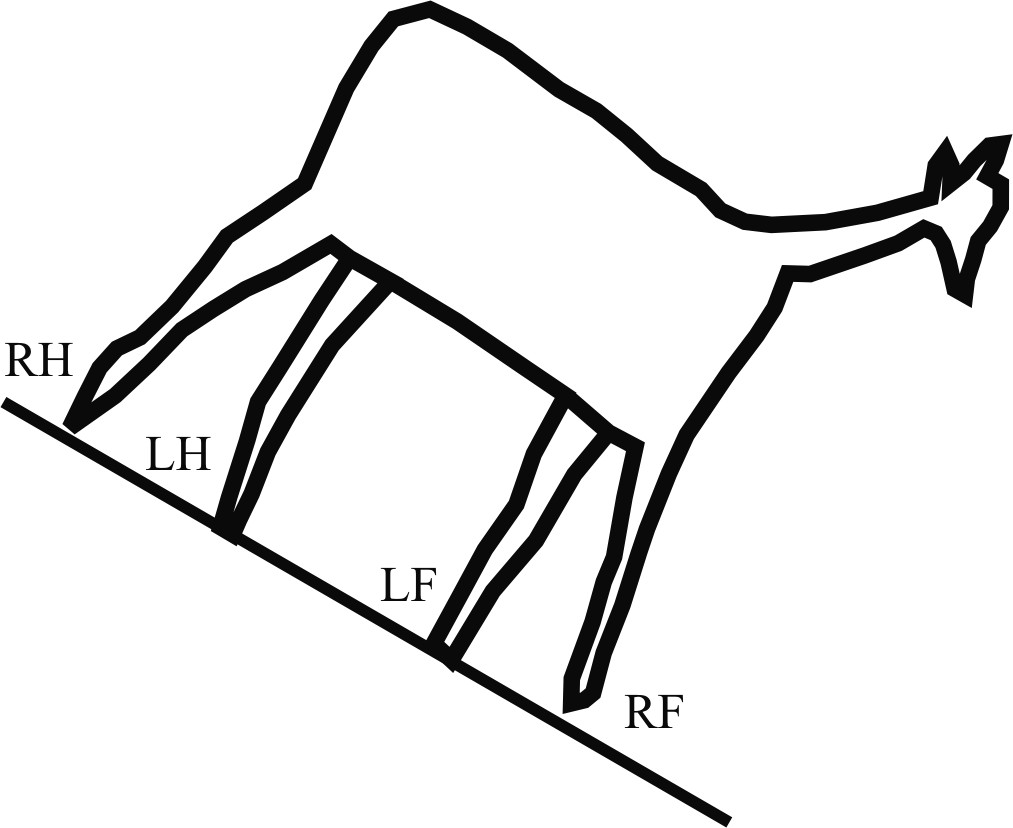


**Supplementary Figure S28**

Supplement: Figure S28 — As Fig. S1 for a prehistoric picture of an antelope from India ( http://www.bradshawfondation.com ). (DOC) [file pone.0049786.s028.doc]

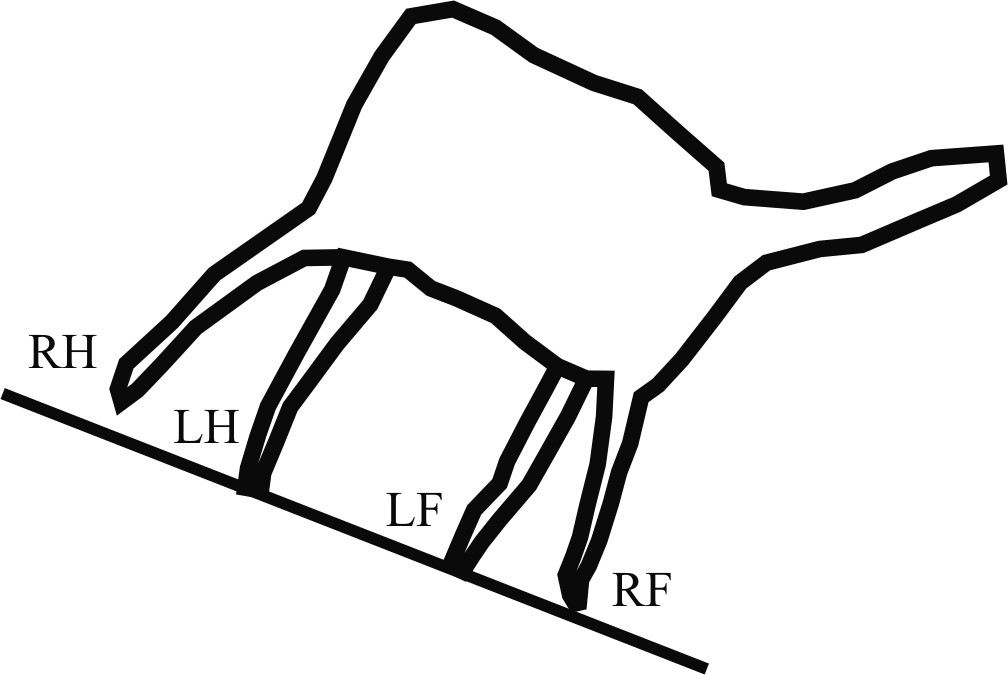


**Supplementary Figure S29**

Supplement: Figure S29 — As Fig. S1 for a prehistoric picture of an antelope from India ( http://www.bradshawfondation.com ). (DOC) [file pone.0049786.s029.doc]

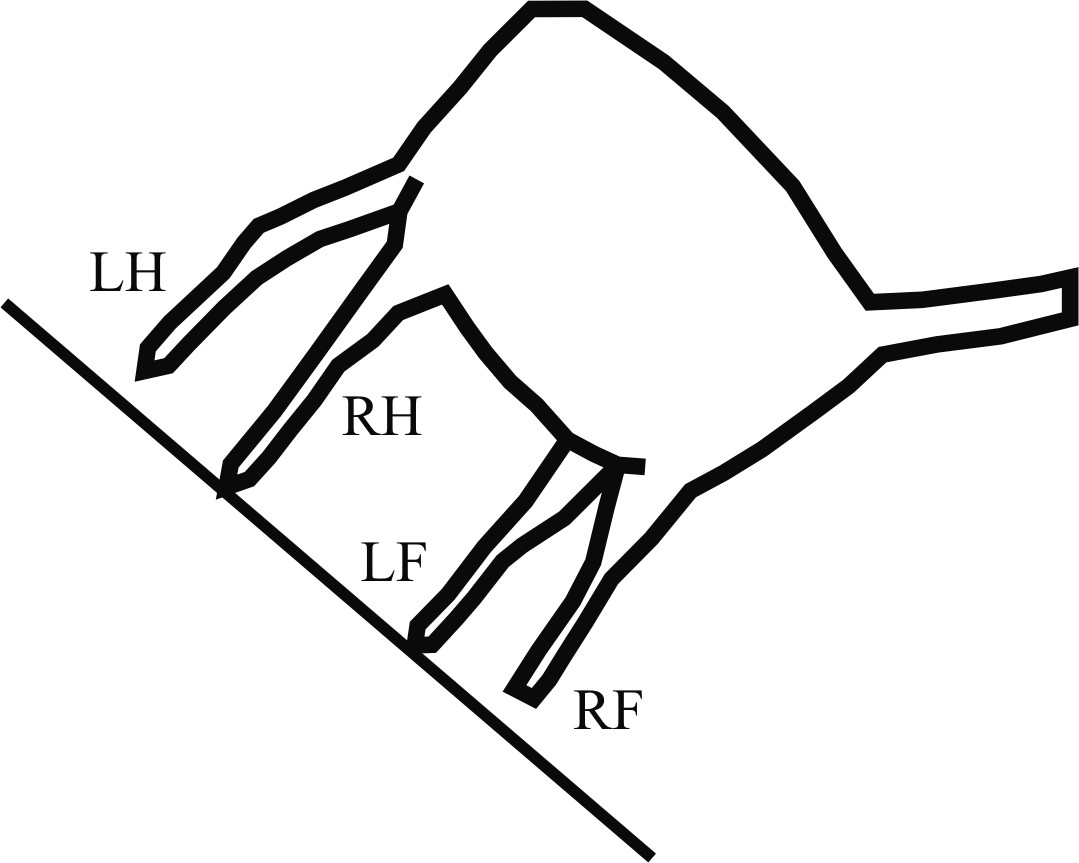


**Supplementary Figure S30**

Supplement: Figure S30 — As Fig. S1 for a prehistoric picture of an antelope from India ( http://www.bradshawfondation.com ). (DOC) [file pone.0049786.s030.doc]

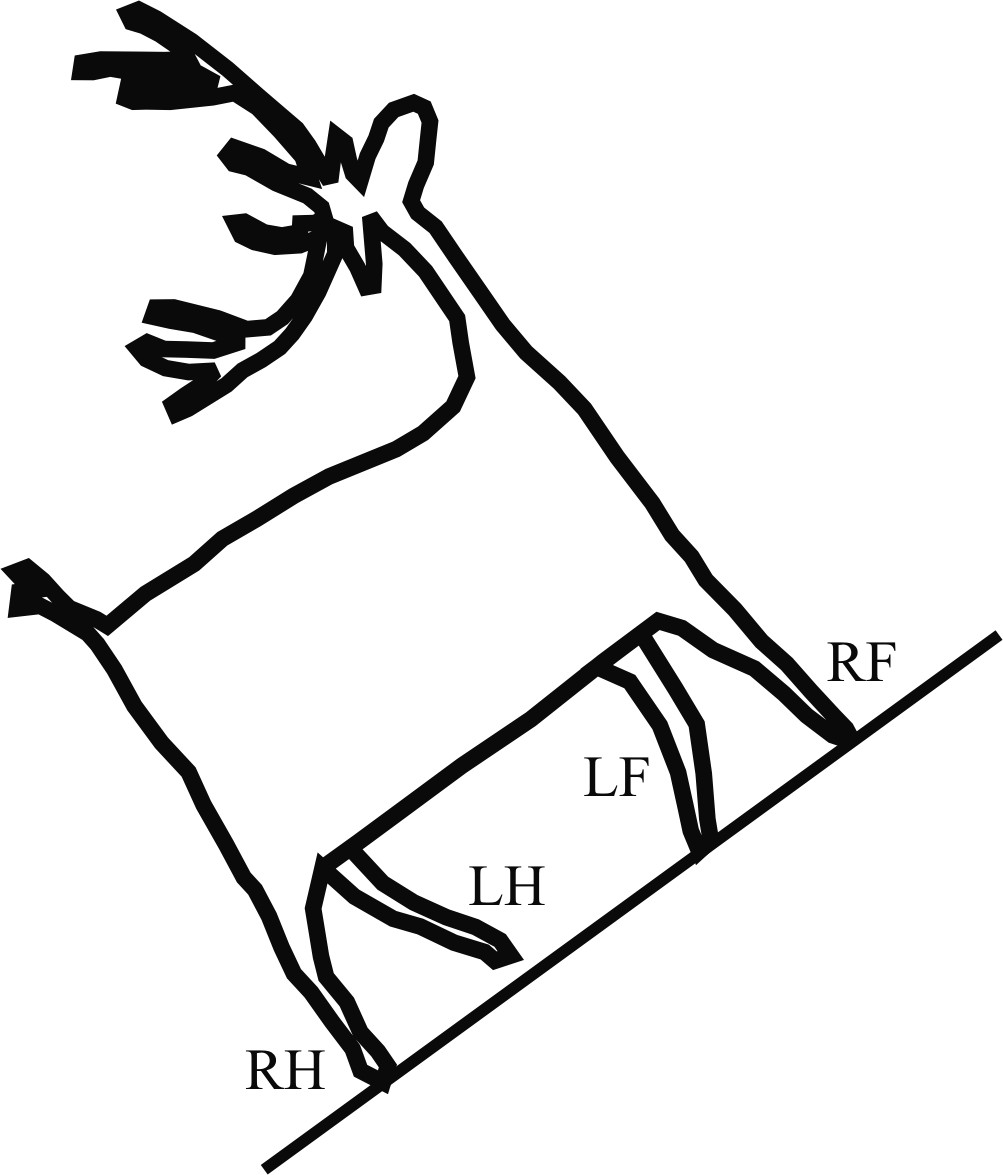


**Supplementary Figure S31**

Supplement: Figure S31 — As Fig. S1 for a prehistoric picture of a deer from India ( http://www.bradshawfondation.com ). (DOC) [file pone.0049786.s031.doc]

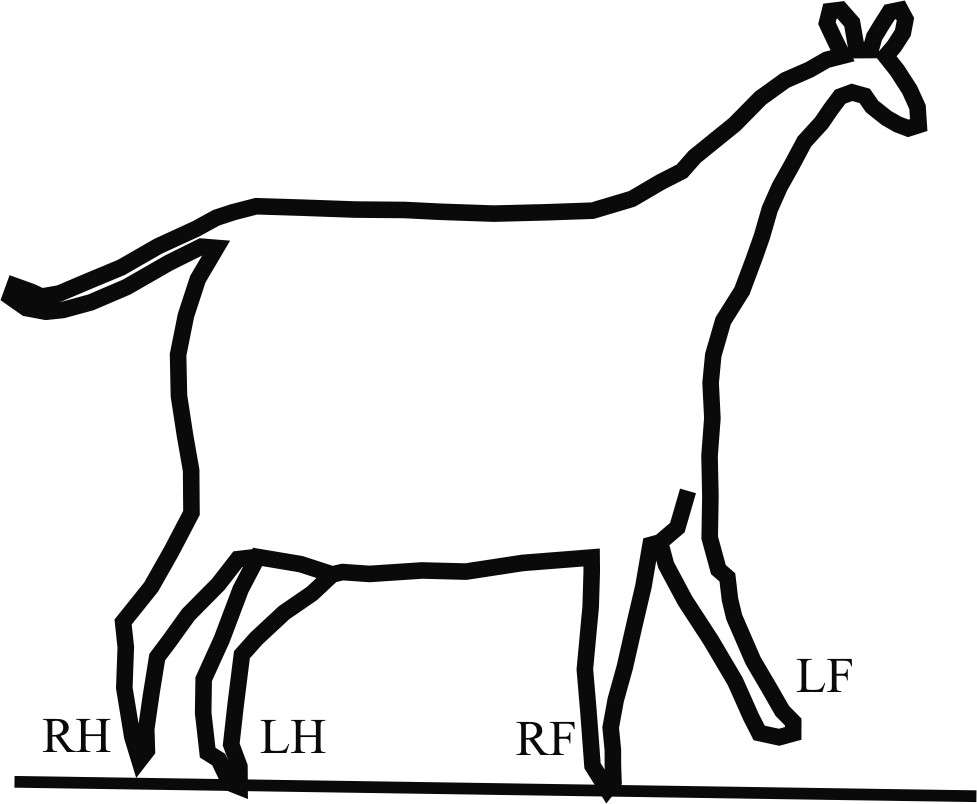


**Supplementary Figure S32**

Supplement: Figure S32 — As Fig. S1 for a prehistoric picture of a quadruped from the Indian Bhimabetaka ( http://bmaks.webs.com/cavepaintings.htm ). (DOC) [file pone.0049786.s032.doc]

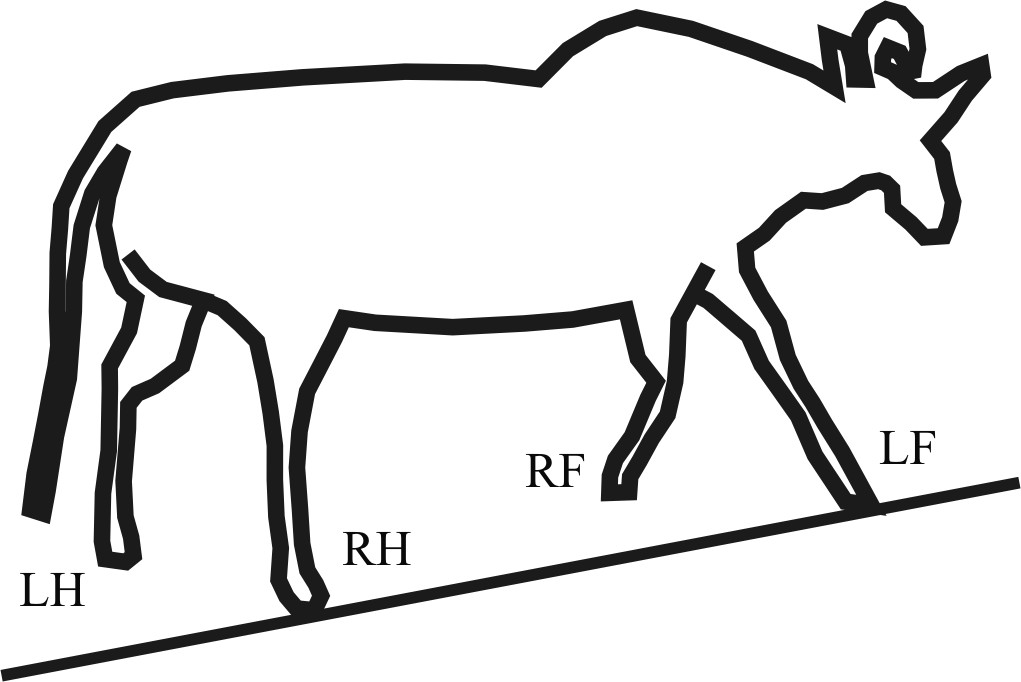


**Supplementary Figure S33**

Supplement: Figure S33 — As Fig. S1 for a prehistoric picture of a bull from India ( http://whc.unesco.org/en/list/925 ). (DOC) [file pone.0049786.s033.doc]

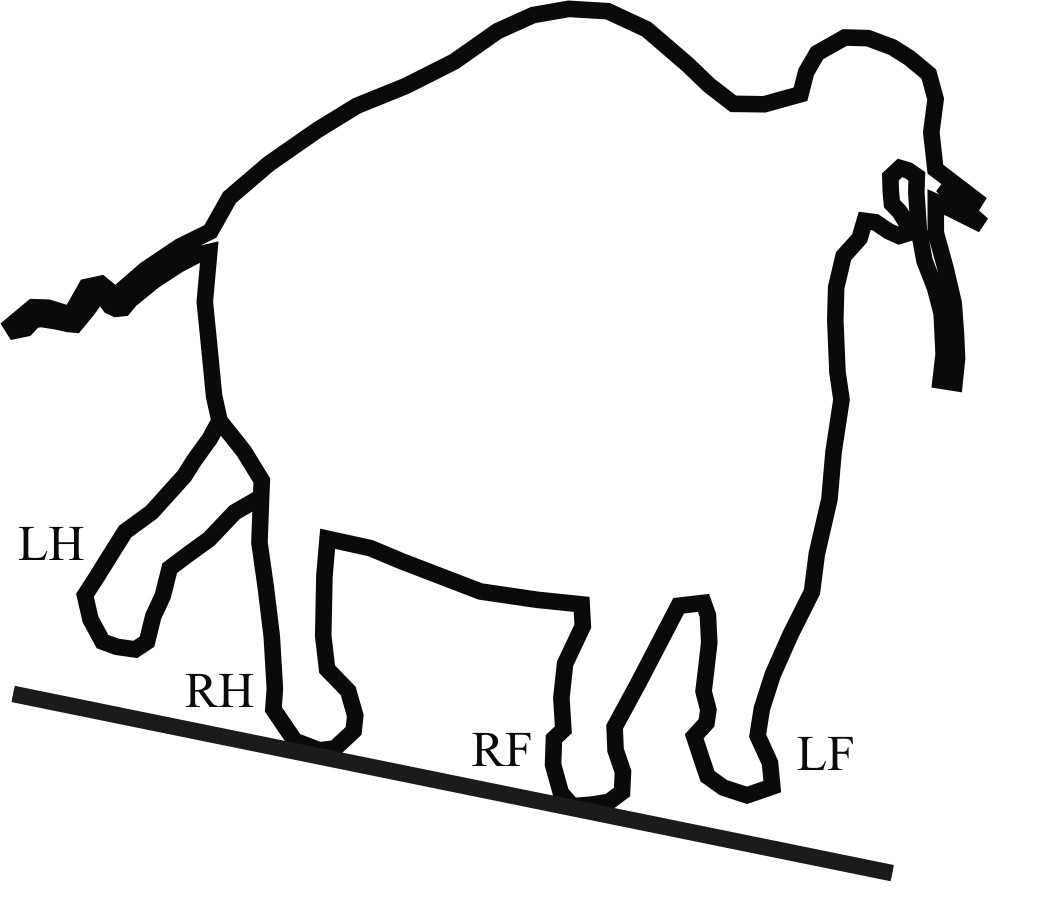


**Supplementary Figure S34**

Supplement: Figure S34 — As Fig. S1 for a prehistoric picture of a mammoth from the Indian Karabad ( http://www.bradshawfondation.com ). (DOC) [file pone.0049786.s034.doc]

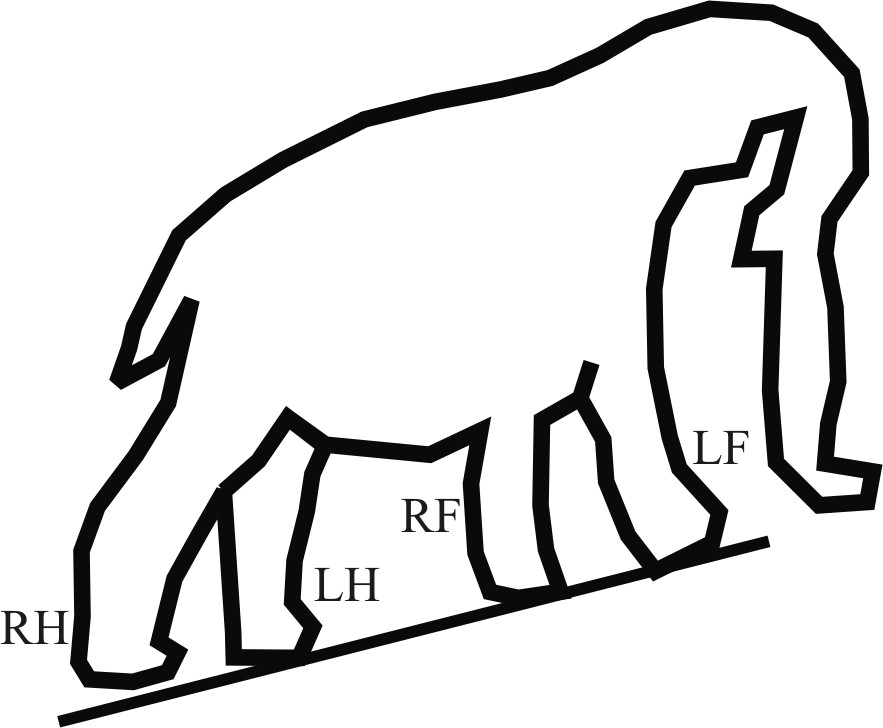


**Supplementary Figure S35**

Supplement: Figure S35 — As Fig. S1 for a prehistoric picture of an elephant from the Indian Bhimabetaka ( http://bmaks.webs.com/cavepaintings.htm ). (DOC) [file pone.0049786.s035.doc]
