# Supplementary material for: Cavemen Were Better at Depicting Quadruped Walking than Modern Artists: Erroneous Walking Illustrations in the Fine Arts from Prehistory to Today
Source: PLoS One. 2012 Dec 5;7(12):e49786. doi: 10.1371/journal.pone.0049786 (PMC3515592; doi:10.1371/journal.pone.0049786)
Supplement: Table S1 — The numbers of all modern (after prehistory) correct (grey cells) and incorrect (white cells) quadruped walking illustrations in the walking matrix. N correct = 334, N incorrect = 627, total N = N correct+N incorrect = 961. The error rate is r = N incorrect/N = 65.2%. (DOC) [file pone.0049786.s036.doc]

**Supplementary Table S1**

|  | a | b | c | d | e | f | g | h |
| --- | --- | --- | --- | --- | --- | --- | --- | --- |
| A | 24 | 9 | 11 | 15 | 1 |  | 2 | 1 |
| B | 69 | 33 | 37 | 108 | 35 | 6 | 11 | 24 |
| C |  | 10 | 11 | 12 |  | 2 | 4 | 3 |
| D | 5 |  | 1 | 12 | 30 | 9 | 6 | 6 |
| E | 13 | 2 | 6 | 11 | 31 | 14 | 52 | 90 |
| F | 26 | 4 |  | 2 | 23 | 22 | 22 | 69 |
| G |  | 5 | 2 | 1 |  | 9 | 15 | 18 |
| H | 12 | 3 |  | 2 | 2 |  | 5 | 3 |
