# Supplementary material for: Cavemen Were Better at Depicting Quadruped Walking than Modern Artists: Erroneous Walking Illustrations in the Fine Arts from Prehistory to Today
Source: PLoS One. 2012 Dec 5;7(12):e49786. doi: 10.1371/journal.pone.0049786 (PMC3515592; doi:10.1371/journal.pone.0049786)
Supplement: Table S2 — The numbers of correct (grey cells) and incorrect (white cells) prehistoric quadruped walking illustrations (see Supporting Information S1) in the walking matrix. N correct = 21, N incorrect = 18, total N = N correct+N incorrect = 39. The error rate is r = N incorrect/N = 46.2%. (DOC) [file pone.0049786.s037.doc]

**Supplementary Table S2**

|  | a | b | c | d | e | f | g | h |
| --- | --- | --- | --- | --- | --- | --- | --- | --- |
| A |  |  |  |  |  |  |  |  |
| B | 3 | 6 |  |  | 3 | 4 |  |  |
| C |  | 3 |  |  |  | 1 |  |  |
| D | 1 |  |  |  |  | 1 |  | 1 |
| E |  |  |  |  |  |  |  |  |
| F |  | 3 |  |  | 3 | 1 |  | 1 |
| G |  |  |  | 1 |  | 1 |  |  |
| H |  | 1 |  |  |  | 1 |  | 4 |
