# Supplementary material for: Cavemen Were Better at Depicting Quadruped Walking than Modern Artists: Erroneous Walking Illustrations in the Fine Arts from Prehistory to Today
Source: PLoS One. 2012 Dec 5;7(12):e49786. doi: 10.1371/journal.pone.0049786 (PMC3515592; doi:10.1371/journal.pone.0049786)
Supplement: Table S4 — The numbers of correct (grey cells) and incorrect (white cells) pre-Muybridgean (after prehistory and prior to 1887) quadruped walking illustrations in the walking matrix. N correct = 45, N incorrect = 227, total N = N correct+N incorrect = 272. The error rate is r = N incorrect/N = 83.5%. (DOC) [file pone.0049786.s039.doc]

**Supplementary Table S4**

|  | a | b | c | d | e | f | g | h |
| --- | --- | --- | --- | --- | --- | --- | --- | --- |
| A | 1 |  | 2 | 4 |  |  |  |  |
| B | 17 | 9 | 7 | 35 | 5 | 3 | 5 | 17 |
| C |  |  | 1 | 1 |  |  | 1 | 1 |
| D |  |  |  |  | 4 |  | 1 | 3 |
| E | 6 |  | 5 | 5 | 6 | 1 | 21 | 53 |
| F | 4 |  |  |  | 2 |  | 9 | 33 |
| G |  | 1 | 1 | 1 |  |  |  | 3 |
| H |  |  |  | 1 | 1 |  | 1 | 1 |
