# Supplementary material for: Cavemen Were Better at Depicting Quadruped Walking than Modern Artists: Erroneous Walking Illustrations in the Fine Arts from Prehistory to Today
Source: PLoS One. 2012 Dec 5;7(12):e49786. doi: 10.1371/journal.pone.0049786 (PMC3515592; doi:10.1371/journal.pone.0049786)
Supplement: Table S5 — The numbers of correct (grey cells) and incorrect (white cells) post-Muybridgean (after 1887) quadruped walking illustrations in the walking matrix. N correct = 289, N incorrect = 397, total N = N correct+N incorrect = 686. The error rate is r = N incorrect/N = 57.9%. (DOC) [file pone.0049786.s040.doc]

**Supplementary Table S5**

|  | a | b | c | d | e | f | g | h |
| --- | --- | --- | --- | --- | --- | --- | --- | --- |
| A | 23 | 9 | 9 | 11 | 1 |  |  | 1 |
| B | 52 | 24 | 29 | 73 | 30 | 3 | 6 | 7 |
| C |  | 10 | 10 | 11 |  | 2 | 3 | 2 |
| D | 5 |  | 1 | 12 | 26 | 9 | 5 | 3 |
| E | 7 | 2 | 1 | 6 | 25 | 13 | 31 | 37 |
| F | 22 | 4 |  | 2 | 21 | 22 | 13 | 36 |
| G |  | 4 | 1 |  |  | 9 | 15 | 15 |
| H | 12 | 1 |  | 1 | 1 | 2 | 4 | 2 |
