# Supplementary material for: Cavemen Were Better at Depicting Quadruped Walking than Modern Artists: Erroneous Walking Illustrations in the Fine Arts from Prehistory to Today
Source: PLoS One. 2012 Dec 5;7(12):e49786. doi: 10.1371/journal.pone.0049786 (PMC3515592; doi:10.1371/journal.pone.0049786)
Supplement: Table S6 — The numbers of correct (grey cells) and incorrect (white cells) cavalry statues in the walking matrix. N correct = 124, N incorrect = 235, total N = N correct+N incorrect = 359. The error rate is r = N incorrect/N = 65.5%. (DOC) [file pone.0049786.s041.doc]

**Supplementary Table S6**

|  | a | b | c | d | e | f | g | h |
| --- | --- | --- | --- | --- | --- | --- | --- | --- |
| A | 14 | 2 | 5 | 10 | 1 |  |  | 1 |
| B | 31 | 7 | 10 | 54 | 22 | 2 | 3 | 1 |
| C |  | 2 | 3 | 2 |  | 1 | 2 | 1 |
| D | 4 |  |  | 8 | 11 | 3 | 1 | 2 |
| E | 4 | 1 | 1 | 4 | 14 | 8 | 14 | 33 |
| F | 18 | 3 |  | 1 | 10 | 8 | 4 | 17 |
| G |  | 2 |  |  |  | 2 | 4 | 7 |
| H | 1 |  |  |  |  |  |  |  |
