# Supplementary material for: Cavemen Were Better at Depicting Quadruped Walking than Modern Artists: Erroneous Walking Illustrations in the Fine Arts from Prehistory to Today
Source: PLoS One. 2012 Dec 5;7(12):e49786. doi: 10.1371/journal.pone.0049786 (PMC3515592; doi:10.1371/journal.pone.0049786)
Supplement: Table S7 — The numbers of correct (grey cells) and incorrect (white cells) two-dimensional (paintings, graphic art, reliefs) quadruped walking illustrations in the walking matrix. N correct = 210, N incorrect = 392, total N = N correct+N incorrect = 602. The error rate is r = N incorrect/N = 65.1%. (DOC) [file pone.0049786.s042.doc]

**Supplementary Table S7**

|  | a | b | c | d | e | f | g | h |
| --- | --- | --- | --- | --- | --- | --- | --- | --- |
| A | 10 | 7 | 6 | 5 |  |  | 2 |  |
| B | 38 | 26 | 27 | 54 | 13 | 4 | 8 | 23 |
| C |  | 8 | 8 | 10 |  | 1 | 2 | 2 |
| D | 1 |  | 1 | 4 | 19 | 6 | 5 | 4 |
| E | 9 | 1 | 5 | 7 | 17 | 6 | 38 | 57 |
| F | 8 | 1 |  | 1 | 13 | 14 | 18 | 52 |
| G |  | 3 | 2 | 1 |  | 7 | 11 | 11 |
| H | 11 | 3 |  | 2 | 2 |  | 5 | 3 |
