# Supplementary material for: Cavemen Were Better at Depicting Quadruped Walking than Modern Artists: Erroneous Walking Illustrations in the Fine Arts from Prehistory to Today
Source: PLoS One. 2012 Dec 5;7(12):e49786. doi: 10.1371/journal.pone.0049786 (PMC3515592; doi:10.1371/journal.pone.0049786)
Supplement: Table S8 — The numbers of correct (grey cells) and incorrect (white cells) horse walking illustrations in the walking matrix. N correct = 244, N incorrect = 585, total N = N correct+N incorrect = 829. The error rate is r = N incorrect/N = 70.6%. (DOC) [file pone.0049786.s043.doc]

**Supplementary Table S8**

|  | a | b | c | d | e | f | g | h |
| --- | --- | --- | --- | --- | --- | --- | --- | --- |
| A | 18 | 8 | 9 | 15 | 1 |  | 2 | 1 |
| B | 32 | 31 | 36 | 104 | 26 | 5 | 14 | 22 |
| C |  | 8 | 11 | 10 |  |  | 2 | 3 |
| D | 3 |  | 1 | 11 | 16 | 9 | 4 | 5 |
| E | 12 | 2 | 6 | 10 | 27 | 14 | 52 | 89 |
| F | 22 | 3 |  | 1 | 17 | 21 | 22 | 67 |
| G |  | 1 | 2 | 1 |  | 6 | 15 | 15 |
| H | 4 | 3 |  | 2 | 1 | 1 | 4 | 2 |
