# Supplementary material for: Cavemen Were Better at Depicting Quadruped Walking than Modern Artists: Erroneous Walking Illustrations in the Fine Arts from Prehistory to Today
Source: PLoS One. 2012 Dec 5;7(12):e49786. doi: 10.1371/journal.pone.0049786 (PMC3515592; doi:10.1371/journal.pone.0049786)
Supplement: Table S9 — The unity walking matrix with number 1 in its every cell. In this case the numbers of correct (grey cells) and incorrect (white cells) quadruped walking illustrations are: N correct = 16, N incorrect = 44, total N = N correct+N incorrect = 60. Then the error rate is r = N incorrect/N = 73.3% corresponding with the pure accident. (DOC) [file pone.0049786.s044.doc]

**Supplementary Table S9**

|  | a | b | c | d | e | f | g | h |
| --- | --- | --- | --- | --- | --- | --- | --- | --- |
| A | 1 | 1 | 1 | 1 | 1 | 1 | 1 | 1 |
| B | 1 | 1 | 1 | 1 | 1 | 1 | 1 | 1 |
| C |  | 1 | 1 | 1 |  | 1 | 1 | 1 |
| D | 1 | 1 | 1 | 1 | 1 | 1 | 1 | 1 |
| E | 1 | 1 | 1 | 1 | 1 | 1 | 1 | 1 |
| F | 1 | 1 | 1 | 1 | 1 | 1 | 1 | 1 |
| G |  | 1 | 1 | 1 |  | 1 | 1 | 1 |
| H | 1 | 1 | 1 | 1 | 1 | 1 | 1 | 1 |
