# Supplementary material for: Cavemen Were Better at Depicting Quadruped Walking than Modern Artists: Erroneous Walking Illustrations in the Fine Arts from Prehistory to Today
Source: PLoS One. 2012 Dec 5;7(12):e49786. doi: 10.1371/journal.pone.0049786 (PMC3515592; doi:10.1371/journal.pone.0049786)
Supplement: Supporting Information S1 — Detailed analysis of prehistoric quadruped walking illustrations. (DOC) [file pone.0049786.s045.doc]

**Supporting Information S1**

**for:**

**Cavemen were better at depicting quadruped walking than modern artists: Erroneous quadruped walking illustrations in the fine arts from prehistory to today**

Gabor Horvath^1^,*, Etelka Farkas, Ildiko Boncz, Miklos Blaho, Gyorgy Kriska

1: Department of Biological Physics, Physical Institute, Eotvos University, Budapest, Hungary

*corresponding author, e-mail address: gh@arago.elte.hu

**This file includes:**

Detailed analysis of prehistoric quadruped walking illustrations

**Detailed Analysis of Prehistoric Quadruped Walking Illustrations**

In this section all figures were drawn by the authors as contours of different walking quadruped animals copied from pictures taken about various prehistoric quadruped illustrations. The websites of the original colour pictures are given in brackets.

We supposed that the legs RH, LF and RF of the cow in Fig. S1 contact the ground, while the leg LH is lifted in the air. Assuming that RF is before LF, this picture falls in the cell *Gf* of the walking matrix (Fig. 1), which means a correct quadruped walking illustration.

The lower half of leg RH of the horse in Fig. S2 is not visible, because it has eroded. Thus, there are two possibilities: (i) All four legs are on the ground, and the picture falls in the cell *Ca* of the walking matrix meaning a correct quadruped walking depinction. (ii) The leg RH is just lifted into the air, representing a correct quadruped walking illustration falling in the cell *Cb* of the walking matrix.

The bull of Fig. S3 is just lifting its leg RF, while its legs RH, LH and Lf are on the ground. Thus this picture falls in the cell *Da* of the walking matrix, meaning an incorrect quadruped walking illustration.

In Fig. S4 the legs RH and LF of the giraffe are on the ground, while the legs LH and RF are in the air. This incorrect quadruped walking depiction falls in the cell *Fb* of the walking matrix.

In Fig. S5 the legs LH and RH of the horse contact the ground, while leg RH is in its lifting and leg LF is in its falling phase. This correct quadruped walking depiction falls in the cell *Bb* of the walking matrix.

The legs LH and RH of the bull in Fig. S6 are on the ground, leg RH is lifted, and leg LF has just been put on the ground. This correct quadruped walking illustration falls in the cell *Bb* of the walking matrix.

In the case of the elephant in Fig. S7 there are three possibilities, depending on the direction of the assumed ground line: (i) In Fig. S7A the legs LH, RH and RF are on the ground, and leg LF is in its falling phase. (ii) In Fig. S7B the leg LF is just lifted, while legs RH, RF and LF are on the ground. (iii) In Fig. S7C legs RH and RF contact the ground, leg LH is lifted, and leg LF has just been put onto the ground. Thus, in case (i), (ii) and (iii) this quadruped walking depiction falls in the cell *Be*, *Cf* and *Bf* of the walking matrix, respectively, all being incorrect.

The legs LH and RF of the cow in Fig. S8 are on the ground, and the leg LF in its falling phase. There are two possibilities for the attitude of leg RH: (i) If RH is just lifted, the picture falls in the cell *Bb* of the walking matrix, meaning a correct quadruped walking depiction. (ii) If RH is on the ground, the picture belongs to the cell *Ba* of the walking matrix, being again a correct quadruped walking illustration.

In Fig. S9 the legs RH and RF of the antelope are in contact with the ground, while the leg LH is in its lifting phase, and leg LF is in its falling phase. Thus this incorrect quadruped walking depiction falls in the cell *Bf* of the walking matrix.

The legs LH, RH and RF of the antelope in Fig. S10 are on the ground, while the leg LF is in the air. This incorrect quadruped walking illustration belongs to the cell *Be* of the walking matrix.

The legs RH and LF of the bull in Fig. S11 are on the ground, and the animal is stepping with its legs LH and RF. This correct quadruped walking illustration falls in the cell *Ff* of the walking matrix.

In Fig. S12 the legs RH and LF of the bull contact the ground, and the animal is stepping with its legs LH and RF. This incorrect quadruped walking illustration falls in the cell *Fh* of the walking matrix.

The horse in Fig. S13 is supported by its legs RH and RF, while the animal is stepping with its legs LH and LF. This incorrect quadruped walking depictions belongs to the cell *Bf* of the walking matrix.

In Fig. S14 the legs RH and RF of the horse are on the ground, and its legs LF and LH are in their lifting phase. This incorrect quaruped walking illustration falls in the cell *Hf* of the walking matrix.

In Fig. S15 the attitudes of the fore legs of the bull are ambiguous. There exist two different possibilities: (i) In Fig. S15A legs RH and RF are on the ground, and legs LH and LF are in falling and lifting phase, respectively. Thus the picture is correct, because it falls in the cell *Hh* of the walking matrix. (ii) In Fig. S15B the animal is supported by its legs RH and LF, while it is stepping with its legs LH and RF. In this case the picture is belongs to the cell *Dh* of the walking matrix and is an incorrect quadruped walking depiction.

The legs RF, LH and RH of the horse in Fig. S16 contact the ground, but the attitude of the right hind hoof hints that leg RH is just lifted. On the other hand, the leg LF is in its falling phase. This correct quadruped walking illustration belongs to the cell *Bb* of the walking matrix.

Figure S17 shows a horse in a posture when its legs LH, RH and RF on the ground, and the leg LF is in its falling phase. Then this picture is correct, because it falls in the cell *Ba* of the walking matrix.

The support of the horse in Fig. S18 is ensured by the legs LH, RH and RF, while the animal is stepping with its leg LF. This incorrect picture belongs to the cell *Be* of the walking matrix.

In Fig. S19 in spite of the strange posture of the giraffe it is obvious that the legs LH, RH and LF support the animal which is stepping with its leg RF. This picture is correct, because it falls in the cell Fe of the walking matrix.

The deer in Fig. S20 is supported by its legs LH, RH and LF, and the animal is stepping with its leg RH, being in its falling phase. This correct picture falls in the cell *Fe* of the walking matrix.

The elephant in Fig. S21 is stepping with its leg RH, while legs RF, LF and LH give its support. This incorrect picture belongs to the cell *Gd* of the walking matrix.

The support of the giraffe in Fig. S22 is ensured by the legs LH and RF, while the leg RH is in its lifting phase, and the leg LF is in its falling phase. This correct quadruped walking illustration falls in the cell *Bb* of the walking matrix.

The buffalo in Fig. S23 is supported by its legs LH and RF, while its legs RH and LF are in their lifting phase. This incorrect picture belongs to the cell *Hb* of the walking matrix.

In Fig. S24 a rhinoceros can be seen, which is supported by the legs LH, RF and LF, while its leg RH is in lifting phase. The cell of this correct picture in the walking matrix is *Cb*.

The deer in Fig. S25 is supported by the legs LH, RH and RF, while its leg LF is in falling phase. This is a correct walking depiction, whose cell in the walking matrix is *Ba*.

In the cow in Fig. S26 the support is ensured by the legs RH and RF. The leg LF is in falling phase, while the leg LH is in lifting phase. This correct picture belongs to the cell *Hh* of the walking matrix.

In Fig. S27 we can see a bull, which is supported by its legs RH and RF. The leg LF is in lifting phase, while the leg LH is in falling phase. This correct picture belongs to the cell *Hh* of the walking matrix.

The antelope in Fig. S28 is supported by the legs LH and LF, while the leg RH is in lifting phase and the leg RF is in falling phase. This incorrect picture belongs to the cell *Fb* of the walking matrix.

The antelope in Fig. S29 steps with the legs RH and RF, while its legs LF and LH are on the ground. This incorrect picture belongs to the cell *Fb* of the walking matrix.

The support of the antelope in Fig. S30 is given by the legs RH and LF. The leg LH is in lifting phase and the leg RF is in falling phase. This correct picture belongs to the cell *Ff* of the walking matrix.

The deer in Fig. S31 is supported by its legs RH and RF, while the legs LH and LF are in lifting and falling phase, respectively. This correct picture belongs to the cell *Hh* of the walking matrix.

In Fig. S32 the quadruped is supported by the legs LH and RF, while the leg RH is in lifting phase and the leg LF is in falling phase. This correct picture belongs to the cell *Bb* of the walking matrix.

The support of the bull in Fig. S33 is given by its legs LF and RH, while both the legs LH and RF are in lifting phase. This incorrect picture belongs to the cell *Df* of the walking matrix.

The mammoth in Fig. S34 is supported by its legs RH and RF, while the leg LH is in lifting phase and the leg LF is in falling phase. This incorrect picture belongs to the cell *Bf* of the walking matrix.

In the case of the elephant in Fig. S35 the support is ensured by the legs LH, LF and RF, while the leg LH is lifted. This correct picture belongs to the cell *Cb* of the walking matrix.
