# Supplementary material for: Cavemen Were Better at Depicting Quadruped Walking than Modern Artists: Erroneous Walking Illustrations in the Fine Arts from Prehistory to Today
Source: PLoS One. 2012 Dec 5;7(12):e49786. doi: 10.1371/journal.pone.0049786 (PMC3515592; doi:10.1371/journal.pone.0049786)
Supplement: Supporting Information S2 — Permission for the use of the colour picture in Figure 2 . (DOC) [file pone.0049786.s046.doc]

**Supporting Information S2**

**for:**

**Cavemen were better at depicting quadruped walking than modern artists: Erroneous quadruped walking illustrations in the fine arts from prehistory to today**

Gabor Horvath^1^,*, Etelka Farkas, Ildiko Boncz, Miklos Blaho, Gyorgy Kriska

1: Department of Biological Physics, Physical Institute, Eotvos University, Budapest, Hungary

*corresponding author, e-mail address: gh@arago.elte.hu

**This file includes:**

Permission for the use of the colour picture in Figure 2

**Permission for the Use of the Colour Picture in Figure 3**

The photographer of the colour picture in Fig. 2 (a prehistoric illustration of an elephant from the Libian Tadrart Acacus), Luca Galuzzi ([http://www.galuzzi.it](http://www.galuzzi.it/)) gave his permission in the following e-mail:

From luca.galuzzi@gmail.com Thu Dec 15 21:57:04 2011

Date: Thu, 15 Dec 2011 21:49:48 +0100

From: Luca Galuzzi <luca.galuzzi@gmail.com>

To: Kriska György <kriska@ludens.elte.hu>

Cc: Gabor Horvath <gh@arago.elte.hu>

Subject: Re: request

Dr. Gyorgy Kriska,

you are welcome to use my picture in your article.

Luca Galuzzi

_________________________

Luca Galuzzi :: www.galuzzi.it

2011/12/15 Kriska Gyorgy <kriska@ludens.elte.hu>

Dear Luca Galuzzi,

we wrote an article entitled

Cavemen depicted quadruped walking more correctly than modern artists:

Erroneous walking illustrations in the fine arts from prehistory to today

by Gabor Horvath, Etelka Farkas, Ildiko Boncz and Gyorgy Kriska

to be submitted to a scientific journal (e.g. the Public Library of Science).

In our paper we would like to use your following photo(s):

picture name: Prehistoric picture of an elephant from the Libian Tadrart Acacus

website: http://www.galuzzi.it

I would like to ask you to send me a written permission via e-mail that we can use the mentioned picture(s) in our paper.

Of course, the website of your picture(s) will be given in the figure legend(s) of our paper.

Thanks in advance, with best wishes: Dr. Gyorgy Kriska (corresponding author)

Group for Methodology in Biology Teaching,

Biological Institute, Eotvos University,

H-1117 Budapest, Pazmany setany 1, Hungary,

e-mail: kriska@ludens.elte.hu
